# Study of Terpenoid Synthesis and Prenyltransferase in Roots of *Rehmannia glutinosa* Based on iTRAQ Quantitative Proteomics

**DOI:** 10.3389/fpls.2021.693758

**Published:** 2021-08-04

**Authors:** Peilei Chen, Xiaoyan Wei, Qianting Qi, Wenjing Jia, Mingwei Zhao, Huina Wang, Yanqing Zhou, Hongying Duan

**Affiliations:** College of Life Sciences, Henan Normal University, Xinxiang, China

**Keywords:** *Rehmannia glutinosa*, iTRAQ, terpenoids, prenyltransferase, qRT-PCR

## Abstract

*Rehmannia glutinosa* has important medicinal value; terpenoid is one of the main active components in *R. glutinosa*. In this study, iTRAQ technique was used to analyze the relative abundance of proteins in roots of *R. glutinosa*, and 6,752 reliable proteins were quantified. GO enrichment results indicated that most proteins were involved in metabolic process or cellular process, 57.63% proteins had catalytic activity, and 65.80% proteins were enriched in membrane-bounded organelle. In roots of *R. glutinosa*, there were 38 KEGG enrichments with significance, more DEPs were found in some pathways, especially the proteasome pathway and TCA cycle with 15.0% DEPs between elongation stage and expansion stage of roots. Furthermore, five KEGG pathways of terpenoid synthesis were found. Most prenyltransferases belong to FPP/GGPP synthase family, involved in terpenoid backbone biosynthesis, and all interacted with biotin carboxylase CAC2. Compared with that at the elongation stage, many prenyltransferases exhibited higher expression at the expansion stage or maturation stage of roots. In addition, eight FPP/GGPP synthase encoding genes were cloned from *R. glutinosa*, namely *FPPS, FPPS1, GGPS, GGPS3, GGPS4, GGPS5, GPPS* and *GPPS2*, introns were also found in *FPPS, FPPS1, GGPS5* and *GGPS2*, and FPP/GPP synthases were more conservative in organisms, especially in viridiplantae, in which the co-occurrence of GPPS or GPPS2 was significantly higher in plants. Further analysis found that FPP/GGPP synthases of *R. glutinosa* were divided into three kinds, GGPS, GPPS and FPPS, and their gene expression was significantly diverse in different varieties, growth periods, or tissues of *R. glutinosa*. Compared with that of *GGPS*, the expression of *GPPS* and *FPPS* was much higher in *R. glutinosa*, especially at the expansion stage and maturation stage. Thus, the synthesis of terpenoids in roots of *R. glutinosa* is intricately regulated and needs to be further studied.

## Introduction

Terpenoids are important secondary metabolites in plants and have important economic value, not only because of their ability to resist diseases and fungus (Keeling and Bohlmann, 2006; Gershenzon and Dudareva, 2007), but also because of their various physiological activities, such as being anti-tumor, anti-inflammatory, and anti-oxidizing (Bohlmann and Zerbe, 2012). In plants, there are two different biosynthetic pathways of terpenoids: MVA pathway in the cytoplasm and MEP pathway in the plastid (Vranová et al., 2013; Henry et al., 2018). The biosynthetic pathway of terpenoids is mainly composed of three stages in plants: the first stage is the formation of intermediates, IPP and DMAPP, which are common precursors of terpenoids and could be interchangeably used in cytoplasm and plastid (Bochar et al., 1999; Lichtenthaler, 1999); the second stage is the synthesis of three direct precursors, GPP (C10), FPP (C15), and GGPP (C20) (Dewick, 2002); and the last stage leads to the formation of terpenoids with different structures and functions by the modification of various enzymes, such as methylation, hydroxylation, glycosylation, and so on (Liu, 2017).

Prenyltransferase is a key enzyme in the biosynthetic pathways of terpenoids and could catalyze the formation of the precursors for monoterpenes, sesquiterpenes, or diterpenes by IPP and DMAPP (Koyama and Ogura, 1999). At present, prenyltransferase encoding genes have been cloned and studied in some plants (Takei et al., 2001; Miyawaki et al., 2004; Brugière et al., 2008), and it was found that prenyltransferase mainly includes FPPS, GGPPS, and GPPS. Under the catalysis of GPPS, GPP is synthesized from one IPP molecule and one DMAPP molecule, and would lead to the synthesis of monoterpene (Wise and Croteau, 1999). Two IPP molecules and one DMAPP molecule are catalyzed to form FPP by FPPS, which would lead to the synthesis of sesquiterpenes and triterpenes (Cane, 1999). Three IPP molecules and one DMAPP molecule are catalyzed to form GGPP by GGPPS, which would lead to the synthesis of diterpenes and tetraterpenes (Martin et al., 2004).

*R. glutinosa* belongs to *Scrophulariaceae Rehmannia* and is rich in sugars, glycosides, alcohols, terpenoids, and other important components; more than 30 kinds of terpenoids have been discovered so far, such as catalpol, motherwort, arachidin, dihydroanthraquinone, glucoside A, D, and so on (Liu et al., 2012). Catalpol is a higher content of terpenoids in *R. glutinosa*, is one of the criteria evaluating medicinal effects of *R. glutinosa* (Wang et al., 2019), and has obvious pharmacological effects on osteoporosis, nervous system, the cardiovascular-cerebrovascular system, and the immune system, as well as having the effect of lowering blood glucose and regulating blood lipid (Huang et al., 2010; Shieh et al., 2011), anti-ionizing radiation (Chen et al., 2013), and anti-inflammation (Han et al., 2012).

At present, multiple omics techniques have been applied in *R. glutinosa* to better understand the formation and development of roots (Li et al., 2015; Sun et al., 2015; Zhou et al., 2018), and to explore the biosynthesis of bioactive components in roots (Sun et al., 2012; Zhou et al., 2016). In order to explore the biosynthetic pathway of terpenoids in *R. glutinosa*, iTRAQ was used for proteomic study on the roots of *R. glutinosa*, furthermore, some prenyltransferase encoding genes were identified and analyzed in *R. glutinosa*.

## Materials and Methods

### Plant Materials

In this experiment, *R. glutinosa* Jinjiu and *R. glutinosa* 85-5 were used. *R. glutinosa* Jinjiu is a new breed recently bred, its main advantages are high yield, stable yield, high quality, and multiple resistances. *R. glutinosa* 85-5 also has high yield and high quality, but is not resistant to water stain and the degradation of some characteristics are more serious. The tuberous roots of *R. glutinosa* were kindly provided by Yongkang Liu and Cuihong Lu in Agricultural Research Institute of Wenxian County, Henan, China.

As described by Duan et al. (2018), tuberous roots of *R. glutinosa* were grown and cultured in test field of Henan Normal University, Xinxiang City, Henan, China. The type of planting soil was loam, planting density of *R. glutinosa* was 30×30 cm, and *R. glutinosa* was managed by conventional field management. Along with the growth and development of *R. glutinosa*, plant experiment materials were taken and studied, especially in the following three growth stages of *R. glutinosa*: the elongate stage (the root is fleshy and cylindrical), expansion stage (the root displays expansion), and the mature stage (the root is spindle-shaped). Roots, stems, and leaves of *R. glutinosa* were taken and stored at −80°C after quick-freezing with liquid nitrogen.

### Preparation and Labeling of Protein Sample

According to the extraction method of phenylic acid (Isaacson et al., 2006) and some improvements, proteins in roots of *R. glutinosa* Jinjiu were extracted. The concentration of protein extracts was determined by BCA method (Smith et al., 1985), protein extracts were detected by 12% SDS-PAGE electrophoresis, and the SDS-PAGE gel was visualized by CBB stain according to Candiano's protocol (Giovanni et al., 2004).

The shotgun comparative proteomic analysis of protein extracts from roots of *R. glutinosa* Jinjiu was studied by the iTRAQ 8-plex experiment. In this study, protein reduce, cysteine block, and digest were performed by FASP method (Jacek et al., 2009). iTRAQ labeling of protein sample was made according to the instruction of iTRAQ 8-plex kit (AB Sciex). After 50 μl tryptic digest (100 μg peptide) and iTRAQ reagent, the extracts were incubated for 2 h at room temperature, the labeled samples were pooled and collected, and then were dried in the vacuum freeze dryer for iTRAQ analysis.

### 2D-LC–MS/MS Analysis of Peptides

The separation of peptides was performed by RPLC using the following method. The dry sample was re-suspended with 100 μl buffer A and the RPLC was employed on the Agilent 1200 HPLC System. The parameter of HPLC column (Agilent) was: Analytical Guard Column 4.6×12.5 mm 5-Micron, Narrow-Bore 2.1×150 mm 5 μm with 215 and 280 nm UV detection. The separation was performed at 0.3 ml/min. Elution of peptides was made with a mobile phase B of 2–90% over 8 min, each segment with 1min interval for 8-52 min was collected into one tube, four or five tubes were mixed with a total of 10 segments, and every segment was dried in a vacuum freeze dryer. Then the freeze-dried polypeptide samples were re-suspended with Nano-RPLC buffer A for RPLC-MS/MS analysis.

The online Nano-RPLC was employed on the Eksigent nanoLC-Ultra™ 2D System (AB SCIEX). The dissolved samples were loaded on C18 nanoLC trap column (100 μm×3 cm, C_18_, 3μm, 150Å) and washed by Nano-RPLC Buffer A (0.1% FA, 2% ACN) at 2 μl/min for 10 min. An elution gradient of 5-35% acetonitrile (0.1% formic acid) in 70 min gradient was used on an analytical ChromXP C18 column (75 μm × 15 cm, C18, 3 μm 120 Å, ChromXP Eksigent) with spray tip. Data acquisition was performed with a Triple TOF 5600 System (AB SCIEX, USA) fitted with a Nanospray III source (AB SCIEX, USA) and a pulled quartz tip as the emitter (New Objectives, USA). For information dependent acquisition, survey scans were acquired in 250 ms and as many as 35 product ion scans were collected if they exceeded a threshold of 150 counts per second (counts/s) with a 2^+^-5^+^ charge-state. The total cycle time was fixed to 2.5 s, a rolling collision energy setting was applied to all precursor ions for CID, and dynamic exclusion was set for ½ of peak width (18 s).

### Identification and Quantification of Proteins

Against transcriptome data of *R. glutinosa*, raw data (.wiff, Sciex) was processed by Protein Pilot Software v. 5.0 (AB SCIEX, USA) using the Paragon algorithm (Shilov et al., 2007).

Furthermore, the experimental data from tandem MS was used to match the theory data to obtain results of protein identification, and protein identification was performed with the search option: emphasis on biological modifications.

### Biological Information Analysis of Reliable Proteins

In this study, the ID of reliable proteins was nonstandard protein ID; it was necessary to extract sequences from the transcriptome data of *R. glutinosa* (Sun et al., 2012). Based on sequence similarity and the sequence of retrieved transcriptome data of *R. glutinosa*, biological information functions of reliable proteins were analyzed using *Arabidopsis thaliana* as background population.

GO analysis was performed by the mainstream database David6.7 (http://david.abcc.ncifcrf.gov/) and QuickGO (http://www.ebi.ac.uk/QuickGO/) to describe GO classification annotation and enrichment analysis of reliable proteins. KEGG Pathway analysis and enrichment analysis results were obtained by mapping protein information to KEGG database. Furthermore, the analysis of PPI is based on string database (http://string.embl.de/) and cytoscape software (http://www.cytoscape.org/).

### Extraction of Genomic DNA and Total RNA

CTAB method was used to extract genomic DNA from roots and leaves of *R. glutinosa* and was performed as described by Duan et al. (2018). The yield and purity of genomic DNA were respectively determined with spectrophotometry at 260 nm, and the integrity of genomic DNA was detected by 0.8% agarose gel electrophoresis.

According to the instruction of RNAiso Plus (TaKaRa, Japan), total RNA was extracted from roots, stems, and leaves of *R. glutinosa*. In this experiment, DNase treatment and phenol-chloroform extraction were used to remove DNase, furthermore total RNA was dissolved in RNase-free dH_2_O. As described in Duan et al. (2019), the integrity of total RNA was detected by 1.0% agarose gel electrophoresis and the yield and purity of total RNA was estimated according to the absorbance at 260 and 280 nm by UV spectrophotometer. Furthermore, the first-strand cDNA of total RNA from roots, stems, or leaves of *R. glutinosa* was synthesized by reverse transcription referring to the instruction of TaKaRa kit (TaKaRa, Japan).

### Cloning and Analysis of Target Genes

Based on transcriptome sequencing results of *R. glutinosa*, the full-length cDNA sequences of target genes were obtained by electronic cloning. According to UTR regions of target genes, primers were designed, and their sequences were listed in Supplementary Table 1. In this study, cDNA and genomic DNA of *R. glutinosa* were respectively used as templates to obtain full-length cDNA or DNA sequence of target genes by PCR amplification. PCR amplification reaction was performed in a volume of 20.0 μl, which was composed of 2.0 μl template, 1.0 μl each primer (10 μM), 10.0 μl 2×Taq plus, and 6.0 μl ddH_2_O. PCR amplification conditions were as follow: pre-denaturation for 5 min at 95°C, followed by 35 cycles (denaturation for 30 s at 94°C, annealing for 30 s and extension for 1 min at 72°C), and the final extension for 10 min at 72°C. Furthermore, the annealing temperatures of FPP/GGP synthase encoding genes were between 51 and 60°C in PCR amplification reaction.

Bioinformatics analysis of target genes were performed using the following method: ORF was analyzed with ORF Finder program (http://www.ncbi.nlm.nih.gov/gorf/gorf.html); amino acid sequence, MW, and other physicochemical properties of putative proteins were predicted by ExPASy proteomics server (http://www.expasy.ch/tools/protparam.html); and functional domain analysis of putative proteins were performed with NCBI-CDS (http://www.ncbi.nlm.nih.gov/Structure/cdd/wrpsb.cgi). In addition, the signal peptide and transmembrane region in putative proteins were detected with Signal P 4.1 (http://www.cbs.dtu.dk/services/SignalP/) or TMHMM (http://www.cbs.dtu.dk/services/TMHMM/), similar sequences of target genes were analyzed by BLASTP (https://blast.ncbi.nlm.nih.gov/Blast.cgi), amino acid sequences of target genes were compared with DNAMAN, the phylogenetic tree of target genes was constructed by MEGA, and the conservative analysis of target genes was performed based on multiple alignments of homologs using string genome (https://string-db.org/).

### Quantitative Real-Time PCR

In this experiment, gene expression of target genes in *R. glutinosa* was detected by qRT-PCR which was conducted by LightCycler 96 Real-time PCR reaction. The cDNA template of qRT-PCR was prepared from total RNA of *R. glutinosa* by PrimeScript RT reagent Kit with gDNA Eraser (Perfect Real Time: TaKaRa, Japan), and the internal reference gene was *TIP41*. Primers used in qRT-PCR were listed in Supplementary Table 1, and were synthesized by YingjieJi Trade Co., Ltd., (Shanghai, China).

The total volume of qRT-PCR reaction system was 20 μl, composed of 2.0 μl cDNA template, 1.0 μl each primer (10 μM), 10 μL SYBR^®^ Green Master Mix, and 6.0 μl ddH_2_O. The reaction conditions of qRT-PCR were as follows: predenaturation for 5 min at 95°C, followed by 45 cycles of denaturation for 5 s at 95°C, and annealing for 20 s at 60°C. The dissolution conditions were 95°C for 15 s, 60°C for 1 min, and 65°C for 15 s, with continuous detection of signals. In addition, the relative expression levels of target genes were normalized and analyzed by the comparative Ct (2^−ΔΔct^) method (Duan et al., 2019).

## Results

### Proteomic Analysis in Roots of *R. glutinosa*

In this study, iTRAQ quantitative proteomic analysis was performed to study the relative abundance of proteins in roots of *R. glutinosa*. Peptides of six or more amino acids in length and with a maximum of two missed cleavages were exclusively considered for the analysis; the original data were obtained according to search results of protein mass spectrometry (ProteomeXchange identifier: PXD025914). After the database search, 8,929 proteins were detected, and 6,752 reliable proteins were obtained according to the screening criteria of reliable protein.

**Figure 1:**
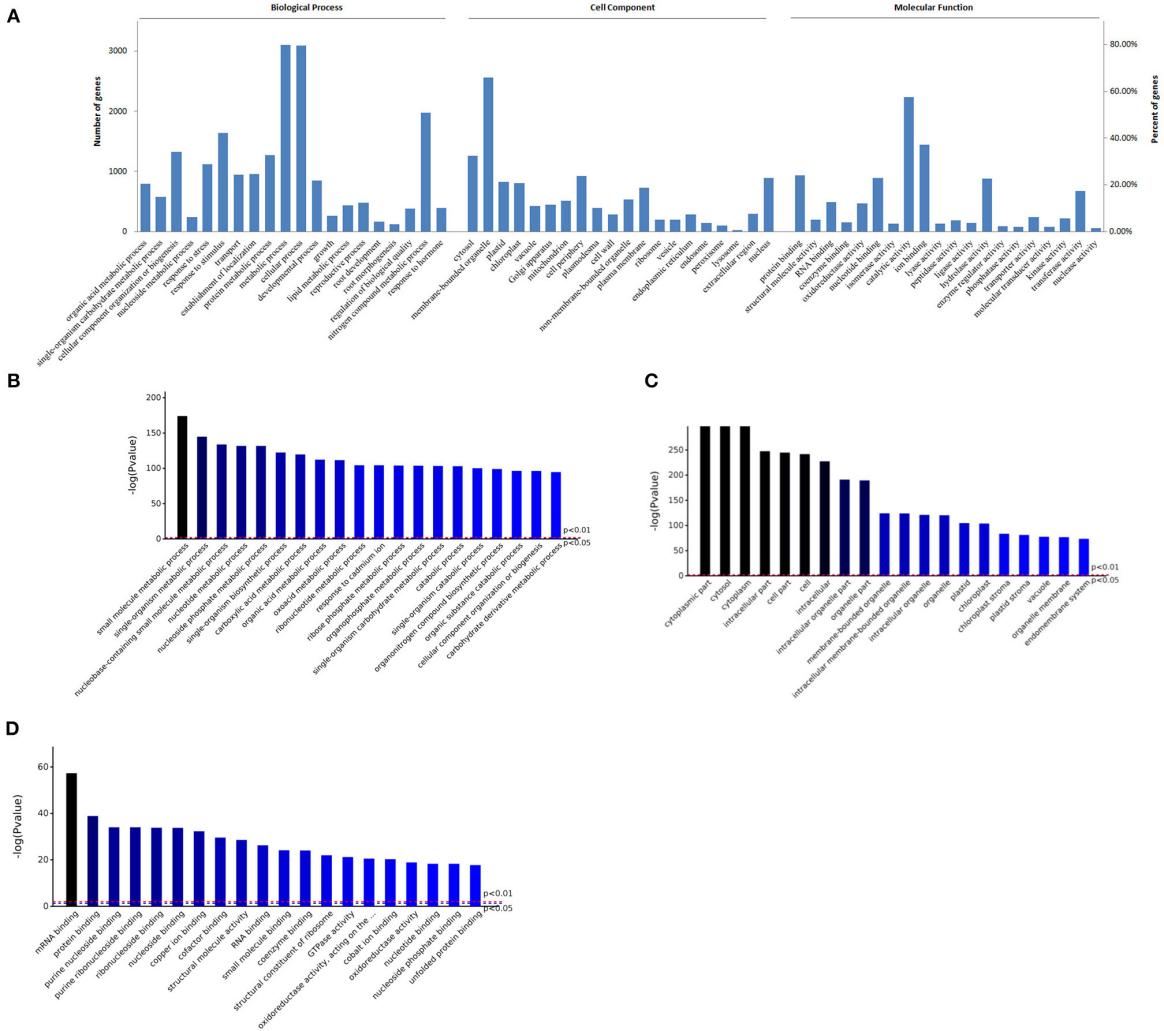
GO enrichment analysis of proteins in roots of *R. glutinosa*. **(A)** GO distribution of proteins, the abscissa represents the entries in each category (Biological Process, Cell Component, Molecular Function), the left and right ordinate represent the number or percent of proteins which are included in each entry. **(B–D)** Represents the top 20 entries in BP, CC, or MF enrichment results, respectively, the abscissa represents the entries, *P* value of red line and blue line is respectively 0.01 or 0.05.

GO enrichment of all proteins were performed. The significant number of BP, CC, and MF was respectively 1704, 360, or 592 (*P* < 0.05) (Supplementary Figure 1). GO function and enrichment analysis were shown in Figure 1, most proteins were involved in metabolic process or cellular process (80% or so), about 42% proteins were in response to stimulus, about 22% proteins were involved in developmental process, and 4 and 3% proteins in root development or root morphogenesis, respectively (Figure 1A). Furthermore, there were some metabolic processes with higher significance (*P* < 0.01), such as small molecule metabolic process, nucleotide metabolic process, and carboxylic acid metabolic process, the biological process response to cadmium ion was also significant, and catabolic process had higher significance (Figure 1B).

In addition, almost every cellular compartment was enriched. Compared to cytosol, about 66% proteins were enriched in membrane-bounded organelle, such as nucleus, chloroplast, mitochondrion, vacuole, ribosome, endoplasmic and reticulum (Figure 1A), and cytosol, membrane-bounded organelle, and chloroplast had higher fold enrichment (*P* < 0.01) (Figure 1C). As shown in Figure 1A, about 58% proteins had catalytic activity, followed by hydrolase activity, transferase activity, or oxidoreductase activity. Furthermore, most proteins belong to ion binding, protein binding, or nucleotide binding, and protein binding or nucleotide binding had higher fold enrichment (*P* < 0.01) (Figure 1D).

### KEGG Pathways in Roots of *R. glutinosa*

**Figure 2:**
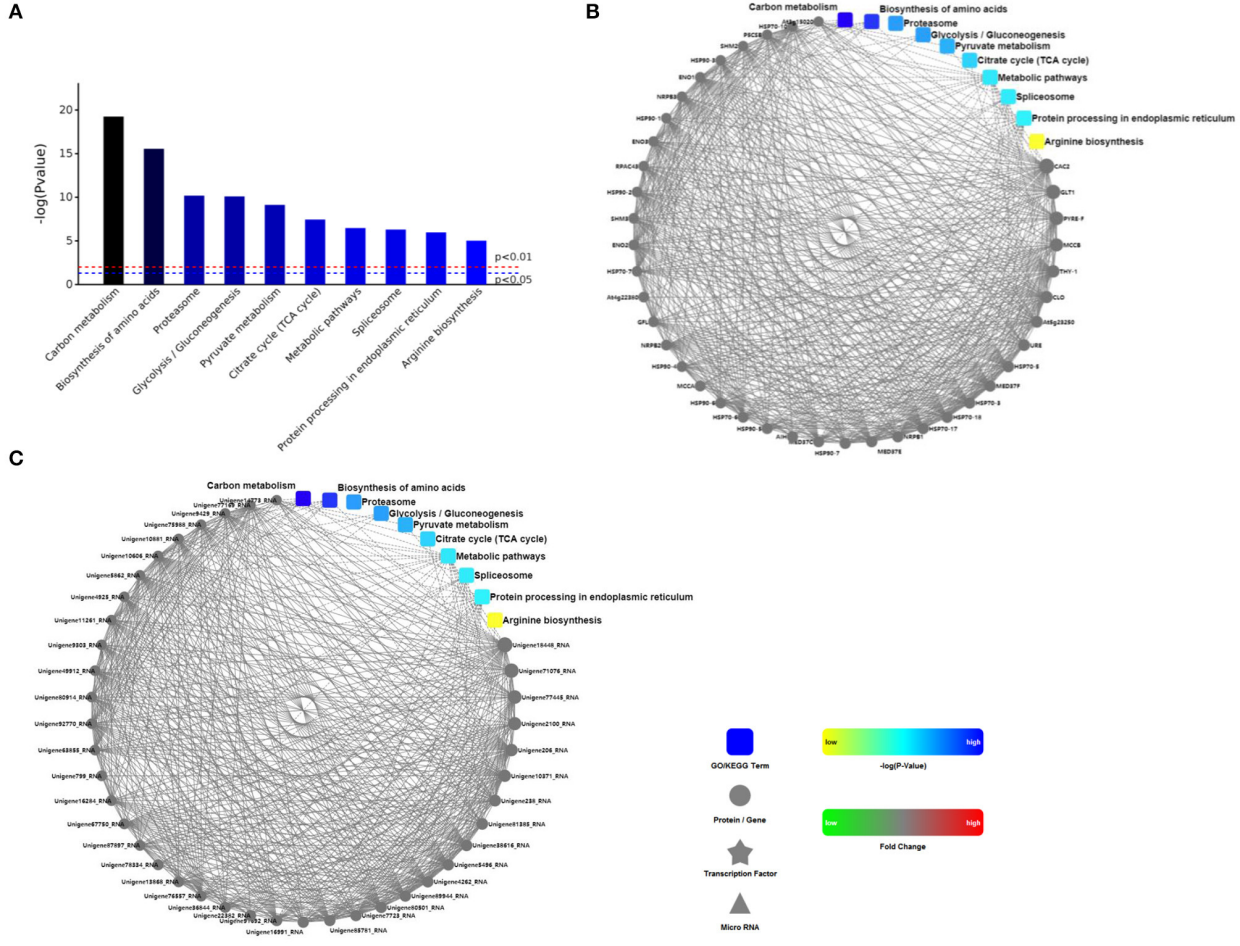
The analysis of KEGG and PPI proteins in roots of *R. glutinosa*. **(A)** The significant top ranking 10 KEGG enrichments, **(B, C)** represents the interaction between the 10 regulatory pathways and the interaction with the protein, is respectively ppigene **(B)** and ppiquery **(C)** of interaction network. *P* value of red line and blue line in **(A)** is respectively 0.01 or 0.05, only when the top of the column is higher than the blue line or the red line, the regulatory pathway is significant (*P* < 0.05) or very significant (*P* < 0.01). Interactive mapping **(B)** or **(C)** is based on dots (gene or protein), polygon (metabolic product), triangle (miRNA), five angle (transcription factor) and rounded rectangle (biological process, cellular localization, molecular functional or signaling pathway) connecting lines, which show the molecular interaction networks and connection mechanism model. The dashed line is without the experimental verification, the solid line is already the relevant verification reports. Furthermore, the dot color change is that red sketch expression is up-regulated, green sketch expression is down-regulated, the rounded rectangle color is that yellow and blue gradient said is from low to high.

Analysis and enrichment results of KEGG pathway in roots of *R. glutinosa* were listed in Supplementary File 1, there were 38 KEGG enrichments with significance (*P* < 0.05) (Supplementary Figure 1), such as metabolic pathways, carbon metabolism, biosynthesis of amino acids, and TCA cycle. The significant top ranking 10 entries (*P* < 0.01) were shown in Figure 2A, the *P* value was very high in carbon metabolism or biosynthesis of amino acids, but was very low in arginine biosynthesis. Furthermore, many proteins were enriched in carbon metabolism, biosynthesis of amino acids, metabolic pathway, spliceosome, and protein processing in endoplasmic reticulum, there were significantly more in the metabolic pathway with 795 proteins (Supplementary File 1). However, less proteins were enriched in proteasome, glycolysis, pyruvate metabolism, TCA cycle, and arginine biosynthesis; only 26 proteins were enriched in arginine biosynthesis (Supplementary File 1).

According to string database and Cytoscape software, PPI in roots of *R. glutinosa* was analyzed (Supplementary File 2), PPI network of proteins in the top ranking 10 KEGG pathways was shown in Figures 2B,C. There were more PPIs in carbon metabolism, metabolic pathway, and protein processing in endoplasmic reticulum, followed by biosynthesis of amino acids and spliceosome, yet PPIs were less in glycolysis, TCA cycle, or arginine biosynthesis, and were not found in proteasome (Figures 2B,C).

During the growth and development of *R. glutinosa*, some proteins were differentially expressed, and DEPs in KEGG enrichments of Figure 2 were further analyzed (Table 1). In some KEGG pathways, there were more DEPs between elongation stage and expansion stage, such as carbon metabolism, biosynthesis of amino acids, proteasome, pyruvate metabolism, and TCA cycle pathway, especially the pathway of proteasome and TCA cycle with 15.0% DEPs (Table 1). More DEPs were also found between other stages, such as proteasome between elongation stage and maturation stage or the pathway of pyruvate metabolism and arginine biosynthesis between expansion stage and maturation stage. However, as listed in Table 1, the percentage of DEPs was relatively lower in some KEGG pathways during the growth and development of *R. glutinosa*, especially spliceosome.

**Table 1:** KEGG enrichment analysis of differentially expressed proteins in roots of *R. glutinosa*.

**Pathway name**	**Pathway ID**	**No. of Proteins**	**Percent of DEPs (%)**		
			**I/E**	**M/E**	**M/I**
Carbon metabolism	ath00940	169	11.8	5.9	7.1
Biosynthesis of amino acids	ath00900	158	11.4	6.3	3.8
Proteasome	ath00945	46	15.2	10.9	2.2
Glycolysis	ath00903	78	7.7	3.9	2.6
Pyruvate metabolism	ath00130	59	10.2	6.8	10.2
TCA cycle	ath00960	45	15.6	2.2	4.4
Metabolic pathways	ath00100	795	8.2	6.3	4.7
Spliceosome	ath00906	105	1.9	0.0	1.0
Protein processing in endoplasmic reticulum	ath00950	113	8.9	9.7	8.0
Arginine biosynthesis	ath00232	26	7.7	7.7	11.5

*E, I, M represents respectively elongation stage, expansion stage, maturation stage of R. glutinosa. I/E, M/E and M/I represent the fold change of protein expression between I stage and E stage, M stage and E stage, or M stage and I stage, respectively. As the fold change is greater than 1.2 or less than 0.83, the expressed protein is considered to be differential (P < 0.05), DEPs indicates differentially expressed proteins*.

### Analysis of Terpenoid Synthesis in *R. glutinosa*

As shown in Supplementary File 1, about 457 proteins were enriched to Biosynthesis of secondary metabolites (ath01110). Some KEGG pathways about the active ingredients of *R. glutinosa* were also found, such as phenylpropanoid, terpene, stilbenoid, steroid, alkaloid, flavonoid, and so on (Table 2). In *R. glutinosa*, there were five KEGG regulatory pathways of terpene synthesis: terpenoid backbone biosynthesis pathway, ubiquinone and other terpenoid-quinone biosynthesis pathway, monoterpenoid biosynthesis pathway, diterpenoid biosynthesis pathway, and sesquiterpenoid and triterpenoid biosynthesis pathway (Table 2). Furthermore, 27 and 15 proteins, respectively, were enriched in terpenoid backbone biosynthesis or other terpenoid-quinone biosynthesis.

**Table 2:** KEGG pathway on some secondary metabolites in roots of *R. glutinosa*.

**No**.	**Pathway name**	**Pathway ID**	**Proteins**	**Count**
1	Phenylpropanoid biosynthesis	ath00940	At3g47040, PER16, BGLU13, CCOAOMT1, CCR2, F13I12.60, 4CLL7, BGLU44, CAD9, PER69, At4g26220, PER73, PER4, PER64, CYP98A3, PER1, MT1, At5g04885, CAD1, BGLU17, TSM1, At5g20950, PER48, F13I12.50, CCR1, CAD5, PER9, PER41, PER26, CAD8, PER52, PER31, PER63, At5g20940, CAD6, CYP73A5, PER42, BGLU32, BGLU16, UGT84A2, PER67, BGLU42, CAD7, BGLU27, 4CL1, CYP84A1, BGLU40, BGLU41, PAL4, PER21, BGLU15, SCPL19	52
2	Terpenoid backbone biosynthesis	ath00900	IPP1, ISPG, FPS1, GGR, DXR, At5g47720, AAT1, FACE1, FTA, At5g58770, SPS2, ISPD, MVD2, ISPE, DXS, At3g32040, SPS3, HMGS, CHLP, At5g27450, GGPPS1, ISPH, At3g29430, ISPF, FPS2, FLCY, At3g20160	27
3	Stilbenoid, diarylheptanoid, and gingerol biosynthesis	ath00945	CCOAOMT1, CYP71B25, At4g26220, CYP98A3, C7A10.20, T5P19_280, CYP73A5, CYP81D1, CYP71B35, CYP71B10, CYP71B24, CYP76G1, CYP82C4, CYP71A18, CYP71B34, CYP71A19	16
4	Limonene and pinene degradation	ath00903	ALDH3H1, CYP71B25, ALDH2B4, ALDH2B7, C7A10.20, T5P19_280, CYP81D1, CYP71B35, CYP71B10, CYP71B24, ALDH3F1, CYP76G1, CYP82C4, CYP71A18, CYP71B34, CYP71A19	16
5	Ubiquinone and other terpenoid- quinone biosynthesis	ath00130	COQ6, PHYLLO, 4CLL7, MENB, VTE3, At4g27270, VTE1, At4g36750, At5g53970, CYP73A5, COQ3, HST, VTE4, COQ5, 4CL1	15
6	Tropane, piperidine and pyridine alkaloid biosynthesis	ath00960	ASP5, ASP2, MHK10.21, PAT, At4g12290, HISN6B, ASP3, At5g53970, At2g29300, At5g06060, ASP1, F23N14_50	12
7	Steroid biosynthesis	ath00100	CYP710A2, 3BETAHSD/D2, SMT1, CAS1, DWF5, 3BETAHSD/D1, SMT2, CYP51G1, FK, DIM, SDP1	11
8	Carotenoid biosynthesis	ath00906	CRTISO, ZDS1, CCD4, ABA2, PDS, NCED3, AAO3, NCED2, Z-ISO, D27, PSY1	11
9	Isoquinoline alkaloid biosynthesis	ath00950	ASP5, ASP2, MHK10.21, PAT, At4g12290, ASP3, At5g53970, ASP1, F23N14_50	9
10	Nicotinate and nicotinamide metabolism	ath00760	QS, At4g14930, QPT, NUDT19, NAPRT1, At1g72880, NAPRT2	7
11	Flavonoid biosynthesis	ath00941	CCOAOMT1, At4g26220, CYP98A3, CYP73A5, DFRA, BAN	6
12	Monoterpenoid biosynthesis	ath00902	SDR1, SDR2b	2
13	Indole alkaloid biosynthesis	ath00901	MES9, MES3	2
14	Diterpenoid biosynthesis	ath00904	GA1	1
15	Sesquiterpenoid and triterpenoid biosynthesis	ath00909	PEN3	1
16	Flavone and flavonol biosynthesis	ath00944	UGT73C6	1
17	Zeatin biosynthesis	ath00908	UGT85A1	1
18	Brassinosteroid biosynthesis	ath00905	CYP734A1	1
19	Caffeine metabolism	ath00232	At2g26230	1

**Figure 3:**
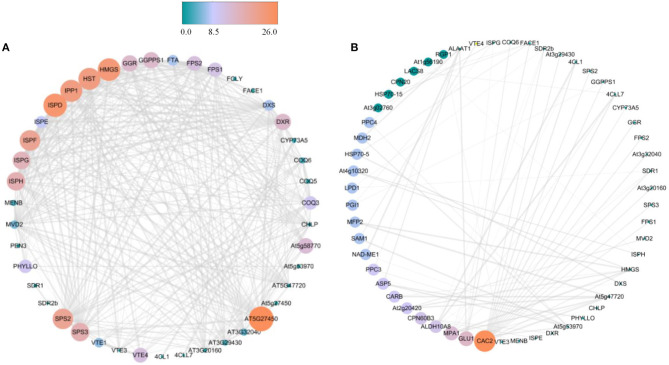
PPI analysis of proteins associated with terpenoid synthesis in *R. glutinosa*. **(A)** PPI analysis between proteins of terpenoid synthesis in *R. glutinosa*, **(B)** PPI analysis between proteins of terpenoid synthesis and others in *R. glutinosa*. The size and color of the circle represents the indegree of PPI, atrovirens circle is low values, but jacinth circle is high values.

Based on string database, PPI analysis of proteins associated with terpenoid synthesis was performed (Supplementary Table 2). Except for AAT1 and TEGA10, most proteins could interact with each other, especially At5G27450, HMGS, ISPD, HST, IPP1, ISPF, and SPS2 (Figure 3A). Further analysis indicated that these terpenoid synthesis proteins could simultaneously interact with other proteins, in which most proteins interacted with CAC2, followed by GLU1 and MPA1, and there was also more interaction with ALDH10A8, PPC3, and ASP5 (Figure 3B).

**Figure 4:**
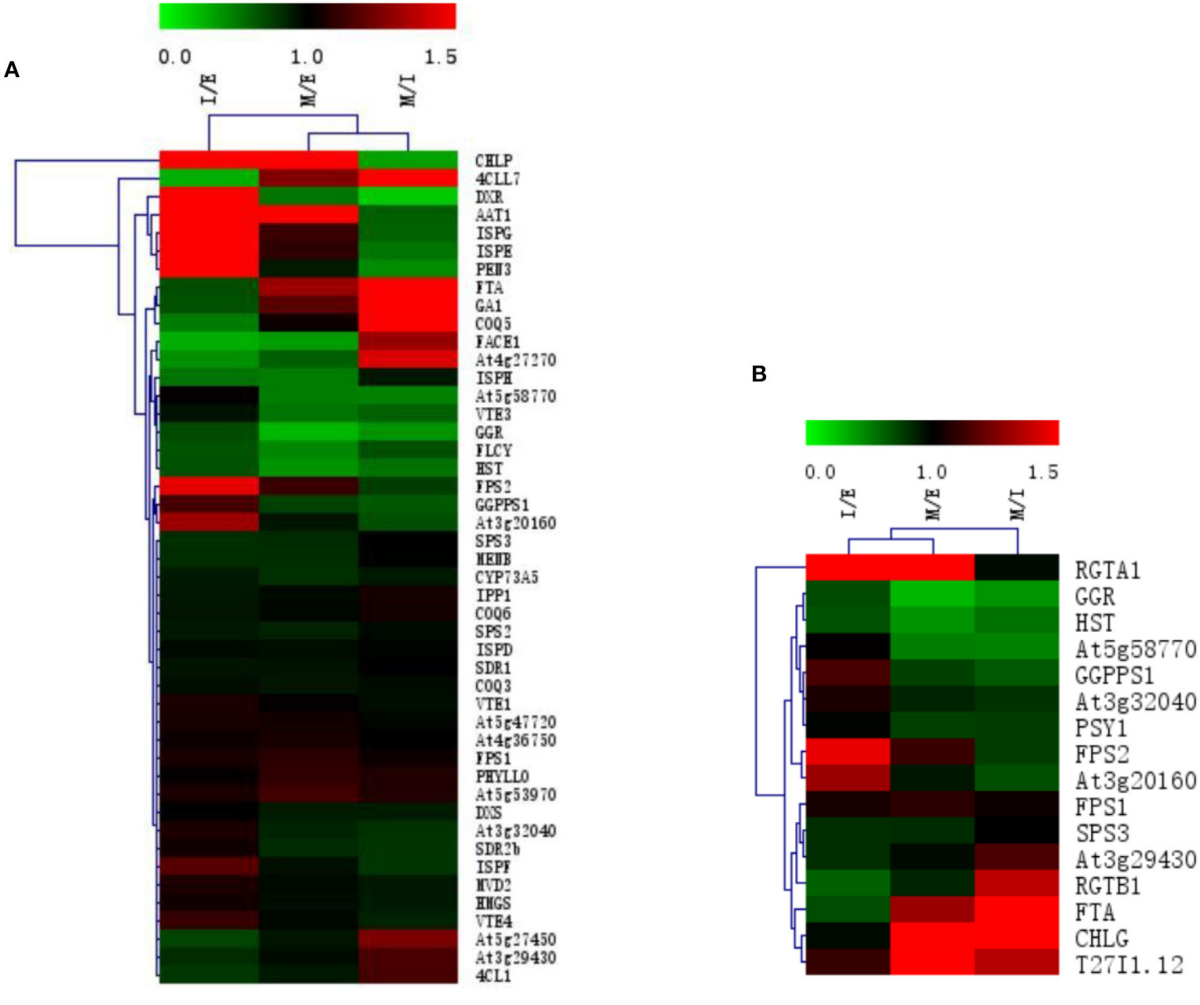
The expression clustering heatmap of some proteins in roots of *R. glutinosa*. **(A)** The expression of proteins associated with terpenoid synthesis in *R. glutinosa*; **(B)** The expression of prenyltransferase in *R. glutinosa*. I/E represents the fold change of protein expression between I stage and E stage of *R. glutinosa*, M/E represents the fold change of protein expression between M stage and E stage of *R. glutinosa*, M/I represents the fold change of protein expression between M stage and I stage of *R. glutinosa*. Green and red color refers to the down or up expression of proteins, respectively. Each line represents the expression of each protein in different groups. Each column represents the expression of all proteins in each group.

During the growth and development of *R. glutinosa*, most proteins associated with terpenoid synthesis can be found differently expressed (*P* < 0.05) (Supplementary Table 3 and Figure 4A). Compared with that at elongation stage, some proteins were up-regulated at expansion stage and maturation stage, such as CHLPA, DXR, AAT1, ISPG, ISPE, PEN3, FPS2, and At3g20160, especially at expansion stag (*P* < 0.05) (Figure 4A). However, the expression of about 26% proteins at elongation stage was significantly higher than that at expansion stage (*P* < 0.05), in which some proteins were also up-regulated at maturation stage, such as 4CLL7, FTA, TGA10, COQ5, FACE1, At4g27270, and ISPH, and the expression of 4CLL7, FTA, and TGA10 at the maturation stage was significantly higher than that at elongation stage (*P* < 0.05) (Figure 4A).

### The Analysis of Prenyltransferase in *R. glutinosa*

As listed in Table 3, these proteins with prenyltransferase activity in *R. glutinosa* were divided into five families, namely FPP/GGPP synthase family, UPP synthase family, protein prenyltransferase family, phytoene/squalene synthase family and UbiA prenyltransferase family, and were enriched in some KEGG pathways, except for T27I1.12, RGTA1, and RGTB1 which belong to protein prenyltransferase subunit family. Further analysis found that most prenyltransferases in *R. glutinosa* belong to FPP/GGPP synthase family, such as FPS1, FPS2, At3g32040, At3g20160, GGPPS1, GGR, and SPS3, which were involved in terpenoid backbone biosynthesis and interacted with biotin carboxylase CAC2. GGPPS1 also interacted with Glucose-6-phosphate isomerase PGI1 (Supplementary Table 3). Furthermore, UPP synthase At5g58770 and protein prenyltransferase subunit alpha FTA were also involved in terpenoid backbone biosynthesis of *R. glutinosa* (Table 3).

**Table 3:** The analysis of prenyltransferase in *R. glutinosa*.

**ID**	**Uniprot gene**	**Description**	**Family**	**Molecular function**	**KEGG pathway**
Unigene5386_RNA	FPS1	Farnesyl pyrophosphate synthase 1	FPP/GGPP synthase	Dimethylallyltranstransferase activity, geranyltranstransferase activity	Terpenoid backbone biosynthesis
Unigene75780_RNA	FPS2	Farnesyl pyrophosphate synthase 2	FPP/GGPP synthase	Dimethylallyltranstransferase activity, geranyltranstransferase activity	Terpenoid backbone biosynthesis
Unigene12015_RNA	At3g32040	Geranylgeranyl pyrophosphate synthase 12	FPP/GGPP synthase	Dimethylallyltranstransferase activity, farnesyltranstransferase activity, geranyltranstransferase activity	Terpenoid backbone biosynthesis
Unigene55090_RNA	At3g20160	Geranylgeranyl pyrophosphate synthase 10	FPP/GGPP synthase	Dimethylallyltranstransferase activity, farnesyltranstransferase activity, geranyltranstransferase activity	Terpenoid backbone biosynthesis
Unigene25249_RNA	GGPPS1	Heterodimeric geranylgeranyl pyrophosphate synthase large subunit 1	FPP/GGPP synthase	Dimethylallyltranstransferase activity, farnesyltranstransferase activity, geranyltranstransferase activity	Terpenoid backbone biosynthesis
Unigene4691_RNA	GGR	Heterodimeric geranylgeranyl pyrophosphate synthase small subunit	FPP/GGPP synthase	Prenyltransferase activity, farnesyltranstransferase activity	Terpenoid backbone biosynthesis
Unigene12226_RNA	SPS3	Solanesyl diphosphate synthase 3	FPP/GGPP synthase	All-trans-nonaprenyl-diphosphate synthase activity, farnesyltranstransferase activity, trans-octaprenyltranstransferase activity	Terpenoid backbone biosynthesis
Unigene14062_RNA	At5g58770	Dehydrodolichyl diphosphate synthase 2	UPP synthase	Dehydrodolichyl diphosphate synthase activity, polyprenyl transferase activity	Terpenoid backbone biosynthesis
Unigene6482_RNA	FTA	Protein farnesyltransferase/ geranylgeranyl transferase type-1 subunit alpha	protein prenyltransferase subunit alpha	Protein geranylgeranyltransferase activity, farnesyltranstransferase activity	Terpenoid backbone biosynthesis
Unigene38410_RNA	T27I1.12	Protein prenylyltransferase superfamily protein	protein prenyltransferase subunit alpha	Protein prenyltransferase activity	
Unigene77883_RNA	RGTA1	Geranylgeranyltransferase type II subunit alpha 1	protein prenyltransferase subunit alpha	Rab geranylgeranyltransferase activity	
Unigene10459_RNA	RGTB1	Geranylgeranyl transferase type-2 subunit beta 1	protein prenyltransferase subunit beta	Rab geranylgeranyltransferase activity	
Unigene69078_RNA	PSY1	Phytoene synthase	phytoene/squalene synthase	Farnesyl-diphosphate farnesyltransferase activity, geranylgeranyl-diphosphate geranylgeranyltransferase activity, squalene synthase activity	Carotenoid biosynthesis
Unigene1711_RNA	CHLG	Chlorophyll synthase, Polyprenyl transferase	UbiA prenyltransferase	Chlorophyll synthetase activity	Porphyrin and chlorophyll metabolism
Unigene11094_RNA	HST	Homogentisate solanesyl transferase	UbiA prenyltransferase	Homogentisate solanyltransferase activity	Ubiquinone and other terpenoid-quinone biosynthesis

In addition, protein expression of prenyltransferase in *R. glutinosa* (shown in Supplementary Table 3 and Figure 4B) showed that the expression of FPS2 and At3g20160 was higher at expansion stage (*P* < 0.05). Compared with that at elongation stage, RGTA1 was continuously up-regulated at expansion stage and maturation stage (*P* < 0.05). FTA, CHLG, and T27I1.12 all exhibited higher expression at maturation stage, but the expressions of GGR and HST continued to decrease at expansion stage and maturation stage, and GGPPS1, At5g58770, and PSY1 were down-regulated at maturation stage (*P* < 0.05).

### Cloning and Analysis of FPP/GGPP Synthase in *R. glutinosa*

In this study, some FPP/GGPP synthase encoding genes were cloned from *R. glutinosa* and their cDNA sequences were submitted to GenBank (Supplementary Figure 2), such as *FPPS* (MG770217), *FPPS1* (MT680921), *GGPS* (MG770218), *GGPS3* (MT680922), *GGPS4* (MT680923), *GGPS5* (MW298275), *GPPS* (MG770219), and *GPPS2* (MW656184). Their full-length CDS sequences were, respectively, 1029, 1050, 1119, 987, 1119, 990, 1272, and 1263 bp (Table 4). Furthermore, intron sequence was found in some FPP/GGPP synthase encoding genes, such as *FPPS, FPPS1, GGPS5*, and *GGPS2*; their DNA sequences were shown in Supplementary Figure 3. The full-length DNA sequence of *FPPS* gene was 3572 bp and was composed of 11 exons and 10 introns, the longest intron was 1073 bp, the shortest intron was 86 bp (Supplementary Figure 3a). As shown in Supplementary Figure 3b, DNA sequence of *FPPS1* gene was 4767 bp with 10 introns; the longest intron was 2015 bp, the shortest intron was 83 bp. DNA sequence of *GPPS2* gene was 3193 bp and was composed of 6 exons and 5 introns, the longest intron was 625 bp and the shortest intron was 81 bp (Supplementary Figure 3d), yet only one intron with 1259 bp was found in *GGPS5* gene (Supplementary Figure 3c).

**Table 4:** Sequence information of FPP/GGPP synthase encoding genes in *R. glutinosa*.

**Gene** **name**	**Accession** **number**	**cDNA length (bp)**	**CDS length (bp)**	**No. of extron**
*FPPS*	MG770217	1,107	1,029	11
*FPPS1*	MT680921	1,345	1,050	11
*GGPS*	MG770218	1,195	1,119	1
*GGPS3*	MT680922	1,315	987	1
*GGPS4*	MT680923	1,317	1,119	1
*GGPS5*	MW298275	1,184	990	2
*GPPS*	MG770219	1,314	1,272	1
*GPPS2*	MW656184	1,737	1,263	6

CDD analysis results showed that FPP/GGPP synthase of *R. glutinosa* is all composed of three functional domains, namely polyprenyl-synt, IspA, and Trans-IPPS-HT, and belongs to Isoprenoid_biosyn_C1 superfamily which share the same isoprenoid synthase fold. The physical and chemical properties of FPP/GGPP synthase in *R. glutinosa* were listed in Table 5. PI was 5.5–6.1 and MW was 40 kD or so. MW of GGPS3 and GPPS5 was about 35 kD, but MW of GPPS and GPPS2 was 46 kD or so. FPP/GGPP synthases were all hydrophilic; only one transmembrane region was detected in FPPS (at 180th−220th) and the subcellular locations of FPP/GGPP synthases were different, for example, FPPS and FPPS1 were located in cytoplasm, GGPS4 and GPPS were located in mitochondrion, and others were located in chloroplast. The secondary structures of FPP/GGPP synthase were generally composed of four parts, namely alpha helix, extended strand, beta turn, and random coil. The proportion of alpha helix was the most (50–65%), followed by random coils (25–33%); the proportion of beta turn was the least (Table 5).

**Table 5:** The analysis of FPP/GGPP synthase in *R. glutinosa*.

**Protein name**	**No. of AA**	**Physico-chemical property**				**Secondary structure (%)**				**Subcellular location**
		**MW (kD)**	**PI**	**Hydrophilicity**	**Transmembrane** **region**	**Alpha** **helix**	**Extended strand**	**Beta turn**	**Random coil**	
FPPS	342	39.93	5.68	Hydrophilicity	180–220	59.36	9.94	5.26	25.44	Cytoplasm
FPPS1	349	39.87	5.62	Hydrophilicity		65.33	6.02	2.87	25.78	Cytoplasm
GGPS	372	40.58	6.14	Hydrophilicity		50.81	12.63	5.91	30.65	Chloroplast
GGPS3	328	35.91	5.86	Hydrophilicity		55.49	9.15	5.18	30.18	Chloroplast
GGPS4	372	40.54	6.14	Hydrophilicity		50.81	6.45	4.84	37.90	Mitochondrion
GGPS5	329	35.91	5.47	Hydrophilicity		54.71	6.69	5.47	33.13	Chloroplast
GPPS	423	46.46	6.14	Hydrophilicity		56.97	11.82	6.62	24.59	Mitochondrion
GPPS2	421	46.57	5.56	Hydrophilicity		60.1	8.79	4.04	27.07	Chloroplast

*AA, amino acid; PI, theoretical isoelectric point; MW, molecular weight*.

**Figure 5:**
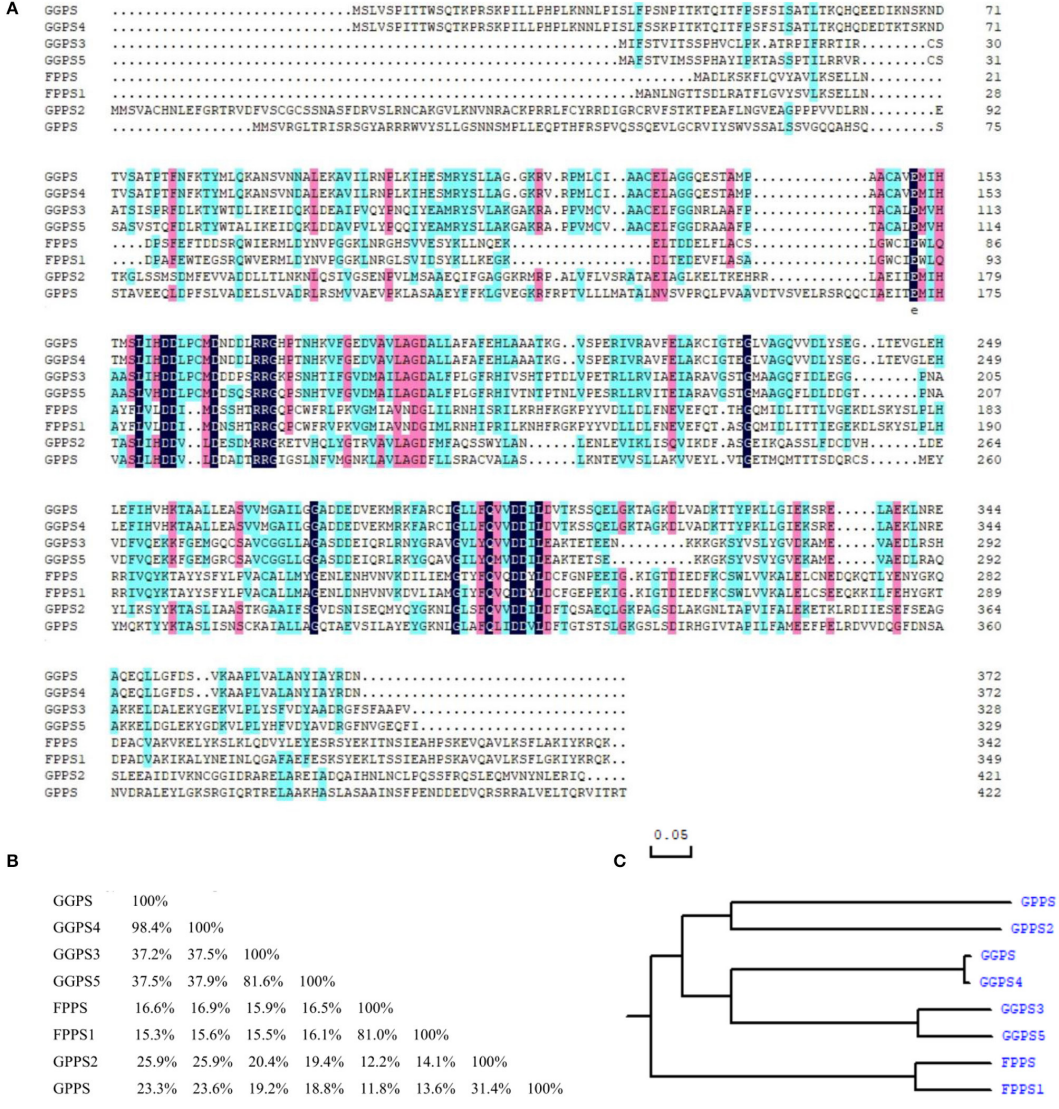
Sequence comparison of FPP/GGPP synthases in *R. glutinosa*. **(A)** Sequence alignment of FPP/GGPP synthase in *R. glutinosa*, **(B)** Homology matrix of FPP/GGPP synthase in *R. glutinosa*, **(C)** Phylogenetic analysis of FPP/GGPP synthase in *R. glutinosa*.

As can be seen in Figures 5A,B, the similarity was lower among FPP/GGPP synthases in *R. glutinosa*, but the identity between their domains was about 37.8%. Further analysis showed that the identity of GGPS and GGPS4 was as high as 98.4% and was also higher (81.6%) between GGPS3 and GGPS5, but the identity of GGPS with GGPS3 or GGPS5 was relatively low, at 38% or so. The similarity of FPPS and FPPS1 was also higher (81.0% identity), yet was 31.4% between GPPS and GPPS2. The identity among other FPP/GGPP synthases was also relatively low. In addition, the Phylogenetic tree of FPP/GGPP synthases in *R. glutinosa* was divided into two branches, FPPS and FPSS1 were clustered into one branch, other synthases were clustered together, in which GGPPS synthases were clustered into one sub-branch, such as GPPS, GPPS3, GPPS4, and GPPS5, while the other sub-branch was composed of GPPS and GPPS2 (Figure 5C).

### The Conservative Analysis of FPP/GGPP Synthase

**Figure 6:**
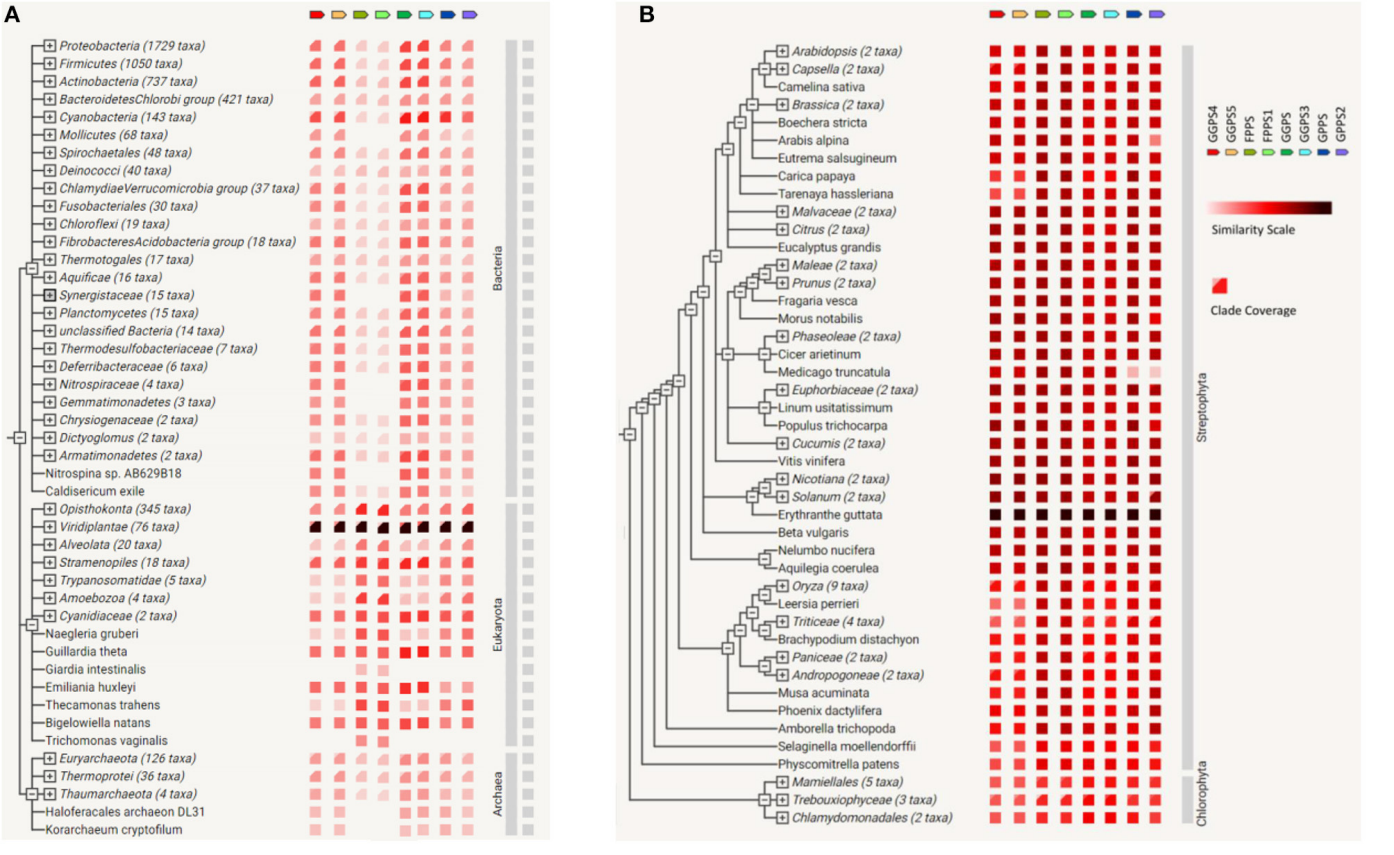
The co-occurrence on FPP/GPP synthase family of *R. glutinosa*. **(A)** Gene co-occurrence of FPP/GPP synthase in the organisom, **(B)** The co-occurrence of FPP/GPP synthase in plants. Similarity Scale: the color denotes, for each gene of FPP/GPP synthase, the similarity of its best hit in a given string genome. Clade Coverage: for groups of genomes that are collapsed in the phylogenetic tree, two distinct colors indicate the lowest and highest similarity observed within that clade.

Based on string genome, gene co-occurrence on FPP/GPP synthase of *R. glutinosa* was analyzed. It was found that FPP/GPP synthase was conservative in organisms and occurred in Archaea, Eukaryota, and Bacteria, in which FPPS, FPPS1, GGPS4, GGPS5, GPPS, or GPPS2 of *R. glutinosa* was more conservative, especially in viridiplantae (Figure 6A). Many genes in viridiplantae appeared to match FPP/GPP synthase of *R. glutinosa* (Table 6), gene co-occurrence for all FPP/GGPP synthases of *R. glutinosa* were all found in 39 species of plants, such as *Arabidopsis thaliana, Brassica rapa, Erythranthe guttata, Glycine max, Gossypium raimondii, Nicotiana tomentosiformis*, and *Vitis vinifera*. 5–7 FPP/GPP synthases were respectively matched in 14 species of plants, in which GGPS and GGPS4 had less co-occurrence. Only 1–2 synthases in 14 species of plants had co-occurrence, and mainly belong to GPPS. In addition, the co-occurrence of FPP/GPP synthase in plants was higher, especially GPPS and GPPS2 (85% co-occurrence), yet the co-occurrence of GGPS, GGPS4 in plants was 57% or so (Table 6).

**Table 6:** The gene co-occurrence on FPP/GPP synthase family of *R. glutinosa*.

**No. of queries matched**	**Plant organism**	**Gene co-occurrence**							
		**FPPS**	**FPPS1**	**GGPS**	**GGP3**	**GGPS4**	**GGPS5**	**GPPS**	**GPPS2**
8	*Amborella trichopoda, Amborella trichopoda, Aquilegia coerulea, Arabidopsis lyrata, Arabidopsis thaliana, Beta vulgaris, Boechera stricta, Brassica oleracea, Brassica rapa, Camelina sativa, Capsella grandiflora, Capsella rubella, Cicer arietinum, Citrus clementina, Citrus sinensis, Cucumis melo, Cucumis sativus, Erythranthe guttata, Eucalyptus grandis, Eutrema salsugineum, Fragaria vesca, Glycine max, Gossypium raimondii, Linum usitatissimum, Malus domestica, Manihot esculenta, Morus notabilis, Nelumbo nucifera, Nicotiana sylvestris, Nicotiana tomentosiformis, Phaseolus vulgaris, Populus trichocarpa, Prunus mume, Prunus persica, Pyrus x bretschneideri, Solanum lycopersicum, Tarenaya hassleriana, Theobroma cacao, Vitis vinifera*	✓	✓	✓	✓	✓	✓	✓	✓
7	*Arabis alpine*	✓	✓	✓	✓	✓	✓		✓
	*Ricinus communis*		✓	✓	✓	✓	✓	✓	✓
	*Solanum tuberosum*	✓	✓	✓	✓	✓	✓		✓
6	*Carica papaya*	✓	✓		✓		✓	✓	✓
	*Medicago truncatula*	✓	✓	✓	✓	✓	✓		
	*Musa acuminate*	✓	✓	✓	✓		✓		✓
	*Oryza glaberrima*	✓	✓		✓		✓	✓	✓
	*Panicum virgatum*	✓	✓		✓		✓	✓	✓
	*Phoenix dactylifera*	✓	✓		✓		✓	✓	✓
	*Setaria italic*	✓	✓		✓		✓	✓	✓
	*Zea mays*	✓	✓		✓		✓	✓	✓
5	*Brachypodium distachyon*		✓		✓		✓	✓	✓
	*Oryza sativa*	✓			✓		✓	✓	✓
4	*Leersia perrieri*	✓	✓					✓	✓
	*Oryza barthii*	✓	✓					✓	✓
	*Oryza brachyantha*	✓	✓					✓	✓
	*Oryza nivara*	✓	✓					✓	✓
	*Oryza punctata*	✓	✓					✓	✓
	*Oryza rufipogon*	✓	✓					✓	✓
	*Sorghum bicolor*	✓	✓					✓	✓
3	*Physcomitrella patens*				✓			✓	✓
	*Triticum aestivum*		✓					✓	✓
2	*Fischerella sp. PCC9339*				✓		✓		
	*Fischerella sp. PCC9431*				✓		✓		
	*Hordeum vulgare*							✓	✓
	*Oryza glumipatula*							✓	✓
	*Oryza meridionalis*							✓	✓
	*Selaginella moellendorffii*							✓	✓
1	*Aegilops tauschii*							✓	
	*Chlamydomonas reinhardtii*							✓	
	*Coccomyxa subellipsoidea*							✓	
	*Fischerella muscicola*				✓				
	*Mastigocladopsis repens*				✓				
	*Ostreococcus lucimarinus*							✓	
	*Ostreococcus tauri*							✓	
	*Triticum urartu*		✓						
Rate of gene co-ocurrence (%)		75.0	78.9	57.9	75.0	56.6	71.1	86.8	84.2

*The symbol indicates the presence of relative genes for FPPS, FPPS1, GGPS, GGP3, GGPS4, GGPS5, GPPS or SPS2 of R. glutinosa*.

Compared with other FPP/GPP synthases of *R. glutinosa*, GGPS and GGPS3 had higher similarity with their relative homologs in Bacteria, the similarity of FPPS or FPPS1 with the relative homologs was also higher in Eukaryota, but was lower in Bacteria and Archaea (Figure 6A). Further analysis showed that all FPP/GGPP synthases of *R. glutinosa* had higher similarity with their homologs in streptophyta, especially in *Erythranthe guttata*, followed by *Nicotiana sylvestris, Nicotiana tomentosiformis, Vitis vinifera, Beta vulgaris, Solanum tuberosum, Beta vulgaris*, and *Arabidopsis thaliana* (Figure 6B; Supplementary Table 4). In addition, FPPS or FPPS1 of *R. glutinosa* had higher similarity with their relative homologs in most plants compared to other synthases (Figure 6B).

### Expression Pattern of FPP/GGPP Synthase Encoding Gene

**Figure 7:**
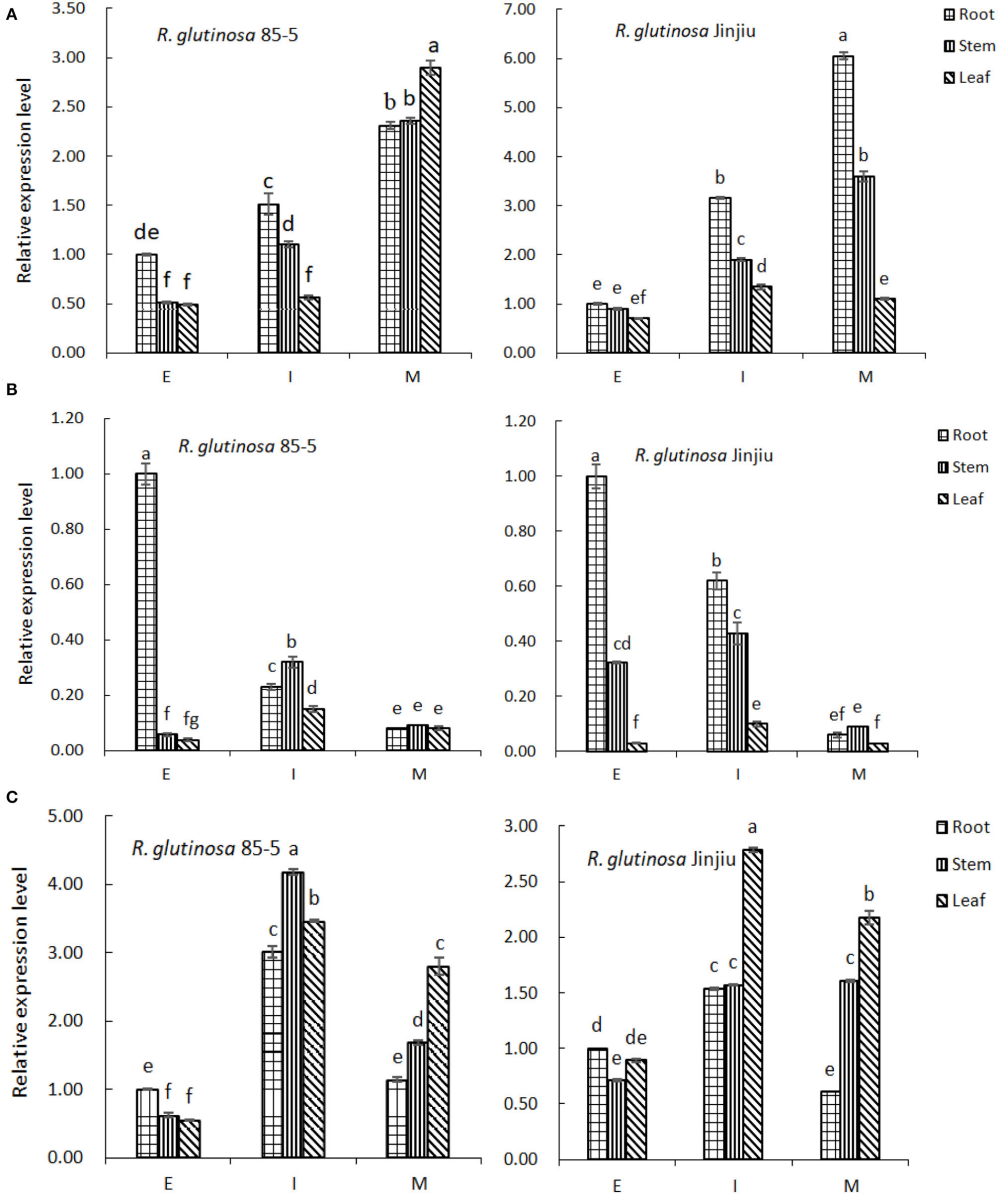
The expression pattern of *FPP/GGPP* gene in *R. glutinosa*. **(A–C)** represents the relative expression of *FPPS, GGPS*, or *GPPS* in *R. glutinosa* 85-5 and *R. glutinosa* Jinjiu, E, I, and M respectively represents elongation stage, expansion stage, and maturation stage of *R. glutinosa*. The error bar is standard error of mean, and the lower letter above the bar indicated the significant difference in root, stem, or leaf among the different growth stages of *R. glutinosa* (*P* < 0.05).

As shown in Figure 7A, along with the growth and development of *R. glutinosa* 85-5, the expression of *FPPS* gene in root, stem, and leaf increased, and was higher at M stage (*P* < 0.05), especially in leaf. Similarly, the expression of *FPPS* gene in *R. glutinosa* Jinjiu also increased continuously and was higher in root and stem at M stage (*P* < 0.05). Furthermore, the expression profile of *FPPS* was obviously varied between *R. glutinosa* 85-5 and *R. glutinosa* Jinjiu, except for that in leaf at M stage, the expression of *FPPS* was generally higher in *R. glutinosa* Jinjiu, and was especially significant in root and stem (Figure 7A).

Along with the growth and development of *R. glutinosa*, the expression of *GGPS* gene in roots decreased continuously (*P* < 0.05), but increased initially and then decreased in stem and leaf, and was higher at I stage (Figure 7B). Compared with that in stem and leaf, the expression of *GGPS* was higher in root of *R. glutinosa* 85-5 at E stage, and was also higher in root of *R. glutinosa* Jinjiu at E–I stage (*P* < 0.05). Although the expression trend of *GGPS* gene was similar between *R. glutinosa* 85-5 and *R. glutinosa* Jinjiu and was both lower at M stage, the expression of *GGPS* was higher at E-I stage of *R. glutinosa* Jinjiu compared with that of *R. glutinosa* 85-5 (Figure 7B).

As can be seen from Figure 7C, the expression of *GPPS* gene in *R. glutinosa* 85-5 first increased and then decreased along with the growth and development of *R. glutinosa*, and was higher at I stage (*P* < 0.05). Compared with that in root, the expression of *GPPS* was significantly higher except in E stage (*P* < 0.05). Similarly, the expression of *GPPS* gene in *R. glutinosa* Jinjiu also first increased and then decreased, reached the highest peak at I stage, and the expression of *GPPS* gene in leaf was higher (*P* < 0.05) (Figure 7C). In addition, the expression of *GGPS* gene was much lower compared with that of *FPPS* and *GPPS*, especially at I–M stage of *R. glutinosa* (*P* < 0.05).

## Discussions

*R. glutinosa* is a perennial herb, its tuberous root is commonly used in Chinese herbal medicines (Duan et al., 2018), can be divided into dried rehamannia root, prepared rehamannia root, and fresh rehamannia root (Lian, 2016). Along with the development of biological technology, omics technique provides a theoretical basis for further study on the molecular mechanism of quality and yield of *R. glutinosa* (Li et al., 2015; Zhou et al., 2018). In this study, iTRAQ quantitative proteomic analysis was used to analyze the relative abundance of proteins in roots of *R. glutinosa*; 8,929 proteins were identified and 6,752 proteins were quantified, but only about 50% reliable proteins in *R. glutinosa* could be further analyzed using *Arabidopsis thaliana* as background population. GO enrichment results indicated that most proteins in roots of *R. glutinosa* were involved in metabolic process or cellular process, 42.26% proteins were in response to stimulus, about 22% proteins were involved in developmental process, and 4.33% were involved in root development. Furthermore, 57.63% proteins had catalytic activity, and 65.80% proteins were enriched in membrane-bounded organelle, especially in nucleus and chloroplast. On account of its high sensitivity, iTRAQ technology is also used to detect proteins in some plants, such as *Beta vulgaris* (Wang, 2017), *Rice* (Qian et al., 2015), *Camelina sativa* (Alvarez et al., 2015), and *Tobacco* (He et al., 2020).

It is well known that roots of *R. glutinosa* are rich in bioactive compounds, such as glycosides, alcohols, terpenoids, and so on (Zhou et al., 2018). In this study, there were 38 KEGG enrichments with significance, such as metabolic pathway, carbon metabolism, biosynthesis of amino acids, and TCA cycle, and more PPIs were found in these pathways. During the growth and development of *R. glutinosa*, more DEPs were found in some KEGG pathways, especially the pathway of proteasome and TCA cycle with 15.0% DEPs between elongation stage and expansion stage of *R. glutinosa*. In addition, some proteins in roots of *R. glutinosa* were also enriched in the secondary metabolism pathway, such as phenylpropanoid biosynthesis, terpenoid backbone biosynthesis, limonene and pinene degradation, and so on. Terpenoids are important secondary metabolites in plants and have a wide range of pharmacological effects and important economic value (Keeling and Bohlmann, 2006; Gershenzon and Dudareva, 2007; Bohlmann and Zerbe, 2012). In *R. glutinosa*, five KEGG terpenoid synthesis pathways were found, many proteins associated with terpenoid synthesis were mainly enriched in terpenoid backbone biosynthesis pathway, but were less in the downstream pathways of terpenoid synthesis, such as monoterpenoid and diterpenoid. Furthermore, most proteins associated with terpenoid synthesis were differently expressed along with the growth and development of *R. glutinosa* and could interact with each other or with CAC2, GLU1, MPA1, and other proteins, suggesting they might participate in the complex metabolic networks.

Prenyltransferase plays an important role in terpenoid synthesis and could catalyze the formation of precursors for monoterpenes, sesquiterpenes, diterpenoids, or other terpenes by IPP and DMAPP (Liu, 2017). In *R. glutinosa*, most prenyltransferases belong to FPP/GGPP synthase family, such as FPS1, FPS2, At3g32040, At3g20160, GGPPS1, GGR, and SPS3, and are involved in terpenoid backbone biosynthesis. All interacted with CAC2. Compared with that at elongation stage, many prenyltransferases exhibited higher expression in roots at expansion stage or maturation stage of *R. glutinosa*. In addition, eight FPP/GGPP synthase encoding genes were cloned from *R. glutinosa*, and introns were found in *FPPS, FPPS1, GGPS5*, and *GGPS2*, in which *FPPS* and *FPPS1* had 10 introns, 5 introns were found in *GPPS2* gene, and only one intron with 1259 bp was found in *GGPS5* gene. Further analysis found that FPP/GPP synthases of *R. glutinosa* were more conservative in organisms, especially in viridiplantae, and the co-occurrence of GPPS or GPPS2 was significantly higher in plants. Furthermore, FPP/GPP synthases of *R. glutinosa* had higher similarity with their homologs in streptophyta, especially in *Erythranthe guttata*.

Although the physical and chemical properties of FPP/GGPP synthases were diverse in *R. glutinosa*, their structures were similarly composed, and these FPP/GGPP synthases of *R. glutinosa* were further divided into three kinds: GGPS, GPPS, and FPPS. As is well known, GGPS is a key enzyme in MEP pathway and is involved in the synthesis of diterpenes and tetraterpenes (Martin et al., 2004), yet FPPS is a key enzyme in MVA pathway and is involved in the synthesis of sesquiterpene and triterpenoids (Cane, 1999). GPPS is mainly involved in MEP pathway, and is related to the synthesis of monoterpene (Wise and Croteau, 1999). Catalpol belongs to monoterpene and is a higher content of terpenoids in *R. glutinosa* (Wang et al., 2019). Compared with that of *GPPS* and *FPPS*, the expression of *GGPS* gene was much lower in *R. glutinosa*, especially at expansion stage and maturation stage, the expression of *GPPS* gene was also higher than that of *FPPS* gene at these stages, which is consistent with the accumulation of catalpol in *R. glutinosa*. Further analysis found that the expression of *FPPS* and *GGPS* in root and stem was higher at expansion stage or maturation stage of *R. glutinosa* Jinjiu, yet *GPPS* gene had high expression in *R. glutinosa* 85-5, which may be related to the characteristics of *R. glutinosa* 85-5 and *R. glutinosa* Jinjiu, which also indicates that gene expression of FPP/GGPP synthase is significantly different in different varieties, growth periods, and tissues of *R. glutinosa*. Similar phenomena were also found in other research (Miyawaki et al., 2004; Fang et al., 2017; Liu, 2017; Duan et al., 2019). In conclusion, prenyltransferase, as a key enzyme in the synthesis pathway of terpenoids, regulates the synthesis direction of terpenoids, but the anabolism network is complex and is regulated by various enzymes. Therefore, the biosynthesis and metabolic regulation mechanism of terpenoids in *R. glutinosa* are complicated and need to be further studied.

## Data Availability Statement

The datasets presented in this study can be found in online repositories (ProteomeXchange, the accession number is PXD025914).

## Author Contributions

HD and PC conceived this experiment. XW, WJ, and HW obtained experiment data. PC and QQ analyzed experiment data and wrote this paper. MZ, PC, and YZ participated in text editing. YZ and HD revised this manuscript. All authors read and approved the final manuscript.

## Conflict of Interest

The authors declare that the research was conducted in the absence of any commercial or financial relationships that could be construed as a potential conflict of interest.

## Publisher's Note

All claims expressed in this article are solely those of the authors and do not necessarily represent those of their affiliated organizations, or those of the publisher, the editors and the reviewers. Any product that may be evaluated in this article, or claim that may be made by its manufacturer, is not guaranteed or endorsed by the publisher.

## Abbreviations

BCA: bicinchonininc acid
BP: biological process
CAC2: Biotin carboxylase
CC: cell component
CID: collision induced dissociation
CTAB: cetyltriethyl ammonium bromide
DMAPP: dimethylallyl diphosphate
DEPs: differentially expressed proteins
MEP: 2-methyl erythritose-4-phosphate
E stage: elongation stage
FASP: filter aided sample preparation
FPP: farnesyl pyrophosphate
FPPS: farnesyl pyrophosphate synthase
I stage: expansion stage
GGPP: geranylgeranyl pyrophosphate
GGPPS: geranylgeranyl pyrophosphate synthase
GO: Gene ontology
GPP: geranyl pyrophosphate
GPPS: geranyl pyrophosphate synthase
ID: identification number
IPP: isopentenyl pyrophosphate
iTRAQ: isobaric tags for relative and absolute quantitation
KEGG: kyoto encyclopedia of genes and genomes
M stage: maturation stage
MF: molecular function
MS: mass spectrometry
MVA: mevalonate
MW: molecular weight
ORF: open reading frame
PGI1: Glucose-6-phosphate isomerase 1
PI: isoelectric point
PPI: protein-protein interaction
qRT-PCR: quantitative real-time PCR
R. glutinosa: Rehmannia glutinosa
TCA cycle: citrate cycle
UTR: untranslated regions.
