# Supplementary material for: Study of Terpenoid Synthesis and Prenyltransferase in Roots of Rehmannia glutinosa Based on iTRAQ Quantitative Proteomics
Source: Front Plant Sci. 2021 Aug 4;12:693758. doi: 10.3389/fpls.2021.693758 (PMC8371554; doi:10.3389/fpls.2021.693758)
Supplement: Supplementary file 1 [file Data_sheet_1.docx]

**Supporting information**

**Table S1 Primers used in this study**

**Table S2 The protein-protein interaction on proteins involved in terpenoid synthesis**

**Table S3 Expression pattern of prenyltransferase and proteins associated with terpenoid synthesis in *R. glutinosa***

E, I, M represents respectively elongation stage, expansion stage, maturation stage of *R. glutinosa*. I/E represents the fold-change of protein expression between I stage and E stage, M/E represents the fold-change of protein expression between M stage and E stage, M/I represents the fold-change of protein expression between M stage and I stage. As the fold-change is greater than 1.2 or less than 0.83, the expressed protein is considered to be differential (p < 0.05).

**Table S4 Some h**[**omologous**](javascript:;)**p**[**rotein**](javascript:;)**s on FPP/GPP synthase family of *R. glutinosa***

The h[omologs](javascript:;) for FPPS, FPPS1, GGPS, GGP3, GGPS4, GGPS5, GPPS, SPS2 of *R. glutinosa* were searched from 76 species of plants, and were all obtained from 39 species of plants. The h[omologs](javascript:;) in the above 8 species had high similarity with FPP/GPP synthase family of *R. glutinosa.*

**Fig. S1 Results of bioinformatics function analysis of proteins in *R. glutinosa***

**Fig. S2 The cDNA and amino acid sequences of FPP/GGPP synthase in *R.glutinosa***

(a), (b), (c), (d), (e), (f), (g) and (h) represents the CDS and amino acid sequences of *FPPS* (MG770217), *FPPS1* (MT680921), *GGPS* (MG770218), *GGPS3* (MT680922), *GGPS4* (MT680923), *GGPS5* (MW298275), *GPPS* (MG770219) and *GPPS2* (MW656184), respectively.

**Fig. S3 The DNA sequences of some FPP/GGPP synthase in *R. glutinosa***

(a), (b), (c) and (d) represents DNA sequences of *FPPS, FPPS1, GGPS5, GPPS2*, respectively, and the intron sequences are shown with the underlined regions.

**Table S1 Primers used in this study**

| **Primer** | **Primer sequence（5’→3’）** |
| --- | --- |
| *GPPS*-F | ATGAT GTCTG TAAGG GGGCT G |
| *GPPS*-R | TTACT TTGTT CTTGT GATGA CTCTC |
| *GPPS2*-F | ATGATGTCAGTGGCATGCCAT |
| *GPPS2*-R | TCATTGTATTCGTTCCAAATTATAG |
| *FPPS*-F | GCCCA TCTCA CTTCC C |
| *FPPS*-R | GTGTT GTTAC TTTTG CCTC |
| *FPPS1*-F | CGTTCAAATTATTCTACCCTGTG |
| *FPPS1*-1R | GATTGCTAAACCCTCCTTGT |
| *GGPS*-F | CAGTC TTCTG AAACA CACAC ATTGG |
| *GGPS*-R | CACCA ATCAA CTATA ACAAA ATC |
| *GGPS3*-F | CGTCAACCCATCTTTCATCT |
| *GGPS3*-R | GACAACTAAATACACTTTGCTCC |
| *GGPS4*-F | TCCATAAATAATTCGCCTAAAT |
| *GGPS4*-R | AAAACACCAATCAACTATAACA |
| *GGPS5*-F | ATGGCTTTTTCAACAGTAATAATGT |
| *GGPS5*-R | TCAAATGAATTGCTCCCCAAC |
| q*GPPS*-F | TCCTG CTATC TAGAG CTTGT GTGGC |
| q*GPPS*-R | ACGCT GATCT GATGT AGTTG TCATT |
| q*GPPS2*-F | TTGACTTCGTGTCTTGTGGGTG |
| q*GPPS2*-R | ACGCCTGTAACAGAACAATCTCC |
| q*FPPS*-F | CTTGGGTGGTGCATTGAGTGG |
| q*FPPS*-R | GAAGTATTAACCCATCATTCACCGC |
| q*FPPS1*-F | ACAATGTGCCTGGAGGGAAGT |
| q*FPPS1*-R | GAAGCCATTCTATGCACCAG |
| q*GGPS*-F | GCCTTCGCGT TCGAGCATT |
| q*GGPS*-R | TAATCCCACTTCAGTCAACCCCTC |
| q*GGPS3*-F | CAATACCTGTGCAGTACCCGAACC |
| q*GGPS3*-R | AGCGCAGGCTGTAGGGAAGG |
| q*GGPS4*-F | AAAGGCAAACTCCGTCAACG |
| q*GGPS4*-R | TGCATAGCATGGGTCTCACTC |
| q*GGPS5*-F | TCAGGACTTATTGGACGGCTCT |
| q*GGPS5*-R | ATAACGCATGGCTTCGTAGAT |
| q*TIP41*-F | TGGCT CAGAG TTGAT GGAGT GCT |
| q*TIP41*-R | CTCTC CAGCA GCTTT CTCGG AGA |

**Table S2 The protein-protein interaction on proteins involved in terpenoid synthesis**

| **Node1** | **Node1_Gene** | **Node1_String** | **Node2** | **Node2_Gene** | **Node2_String** | **Score** |
| --- | --- | --- | --- | --- | --- | --- |
| Q8H0S9 | MPA1 | 3702.AT1G63770.5 | Q9FIK7 | At5g47720 | 3702.AT5G47720.2 | 920 |
| Q8H0S9 | MPA1 | 3702.AT1G63770.5 | P54873 | HMGS | 3702.AT4G11820.2 | 998 |
| Q8H0S9 | MPA1 | 3702.AT1G63770.5 | Q9M2E2 | SDR1 | 3702.AT3G61220.2 | 470 |
| Q8H0S9 | MPA1 | 3702.AT1G63770.5 | Q9M0X9 | 4CLL7 | 3702.AT4G05160.1 | 507 |
| Q8H0S9 | MPA1 | 3702.AT1G63770.5 | Q42524 | 4CL1 | 3702.AT1G51680.1 | 412 |
| Q8H0S9 | MPA1 | 3702.AT1G63770.5 | Q9ZUH5 | SDR2b | 3702.AT2G24190.2 | 470 |
| Q9M5K3 | LPD1 | 3702.AT1G48030.1 | Q9ZUH5 | SDR2b | 3702.AT2G24190.2 | 446 |
| Q9M5K3 | LPD1 | 3702.AT1G48030.1 | Q9M2E2 | SDR1 | 3702.AT3G61220.2 | 446 |
| Q8H103 | PGI1 | 3702.AT4G24620.1 | F4K0E8 | ISPG | 3702.AT5G60600.1 | 472 |
| Q8H103 | PGI1 | 3702.AT4G24620.1 | P34802 | GGPPS1 | 3702.AT4G36810.1 | 463 |
| Q9ZPI5 | MFP2 | 3702.AT3G06860.1 | Q9FIK7 | At5g47720 | 3702.AT5G47720.2 | 998 |
| Q9ZPI5 | MFP2 | 3702.AT3G06860.1 | P54873 | HMGS | 3702.AT4G11820.2 | 900 |
| F4IYF8 | At3g02760 | 3702.AT3G02760.1 | F4K0E8 | ISPG | 3702.AT5G60600.1 | 680 |
| O04983 | CAC2 | 3702.AT5G35360.3 | P54873 | HMGS | 3702.AT4G11820.2 | 648 |
| O04983 | CAC2 | 3702.AT5G35360.3 | Q9ZUH5 | SDR2b | 3702.AT2G24190.2 | 669 |
| O04983 | CAC2 | 3702.AT5G35360.3 | Q9FIK7 | At5g47720 | 3702.AT5G47720.2 | 944 |
| O04983 | CAC2 | 3702.AT5G35360.3 | Q9LIA0 | At3g29430 | 3702.AT3G29430.1 | 649 |
| O04983 | CAC2 | 3702.AT5G35360.3 | Q42524 | 4CL1 | 3702.AT1G51680.1 | 774 |
| O04983 | CAC2 | 3702.AT5G35360.3 | Q76FS5 | SPS2 | 3702.AT1G17050.1 | 649 |
| O04983 | CAC2 | 3702.AT5G35360.3 | P34802 | GGPPS1 | 3702.AT4G36810.1 | 649 |
| O04983 | CAC2 | 3702.AT5G35360.3 | Q9M0X9 | 4CLL7 | 3702.AT4G05160.1 | 774 |
| O04983 | CAC2 | 3702.AT5G35360.3 | P92994 | CYP73A5 | 3702.AT2G30490.1 | 589 |
| O04983 | CAC2 | 3702.AT5G35360.3 | Q39108 | GGR | 3702.AT4G38460.1 | 649 |
| O04983 | CAC2 | 3702.AT5G35360.3 | Q43315 | FPS2 | 3702.AT4G17190.1 | 649 |
| O04983 | CAC2 | 3702.AT5G35360.3 | Q9LHR4 | At3g32040 | 3702.AT3G32040.1 | 649 |
| O04983 | CAC2 | 3702.AT5G35360.3 | Q9M2E2 | SDR1 | 3702.AT3G61220.2 | 669 |
| O04983 | CAC2 | 3702.AT5G35360.3 | Q9LJY2 | At3g20160 | 3702.AT3G20160.1 | 649 |
| O04983 | CAC2 | 3702.AT5G35360.3 | Q5HZ00 | SPS3 | 3702.AT2G34630.2 | 649 |
| O04983 | CAC2 | 3702.AT5G35360.3 | Q09152 | FPS1 | 3702.AT5G47770.1 | 649 |
| O82662 | At2g20420 | 3702.AT2G20420.1 | P54873 | HMGS | 3702.AT4G11820.2 | 816 |
| O82662 | At2g20420 | 3702.AT2G20420.1 | F4JCU3 | MVD2 | 3702.AT3G54250.1 | 639 |
| O82662 | At2g20420 | 3702.AT2G20420.1 | Q9FIK7 | At5g47720 | 3702.AT5G47720.2 | 839 |
| Q9SIU0 | NAD-ME1 | 3702.AT2G13560.1 | F4I7I0 | ALAAT1 | 3702.AT1G17290.1 | 943 |
| Q9SIU0 | NAD-ME1 | 3702.AT2G13560.1 | Q9FN30 | At5g53970 | 3702.AT5G53970.1 | 446 |
| Q84VW9 | PPC3 | 3702.AT3G14940.1 | F4I7I0 | ALAAT1 | 3702.AT1G17290.1 | 523 |
| Q84VW9 | PPC3 | 3702.AT3G14940.1 | Q9LY74 | VTE3 | 3702.AT3G63410.1 | 400 |
| Q84VW9 | PPC3 | 3702.AT3G14940.1 | Q9FN30 | At5g53970 | 3702.AT5G53970.1 | 523 |
| P23686 | SAM1 | 3702.AT1G02500.1 | Q9FN30 | At5g53970 | 3702.AT5G53970.1 | 908 |
| P23686 | SAM1 | 3702.AT1G02500.1 | Q9ZSK1 | VTE4 | 3702.AT1G64970.1 | 633 |
| Q9S795 | ALDH10A8 | 3702.AT1G74920.1 | Q9M2E2 | SDR1 | 3702.AT3G61220.2 | 420 |
| Q9S795 | ALDH10A8 | 3702.AT1G74920.1 | Q9ZUH5 | SDR2b | 3702.AT2G24190.2 | 420 |
| Q9S795 | ALDH10A8 | 3702.AT1G74920.1 | Q9M0X9 | 4CLL7 | 3702.AT4G05160.1 | 935 |
| Q9S795 | ALDH10A8 | 3702.AT1G74920.1 | Q42524 | 4CL1 | 3702.AT1G51680.1 | 944 |
| Q9ZNZ7 | GLU1 | 3702.AT5G04140.2 | F4K0E8 | ISPG | 3702.AT5G60600.1 | 424 |
| Q9ZNZ7 | GLU1 | 3702.AT5G04140.2 | Q9FN30 | At5g53970 | 3702.AT5G53970.1 | 762 |
| Q9ZNZ7 | GLU1 | 3702.AT5G04140.2 | F4J6I6 | COQ6 | 3702.AT3G24200.2 | 599 |
| Q9ZNZ7 | GLU1 | 3702.AT5G04140.2 | Q8RX88 | FACE1 | 3702.AT4G01320.1 | 694 |
| Q9ZNZ7 | GLU1 | 3702.AT5G04140.2 | Q9FIK7 | At5g47720 | 3702.AT5G47720.2 | 559 |
| Q9ZNZ7 | GLU1 | 3702.AT5G04140.2 | F4I7I0 | ALAAT1 | 3702.AT1G17290.1 | 826 |
| Q9ZNZ7 | GLU1 | 3702.AT5G04140.2 | Q9CA67 | CHLP | 3702.AT1G74470.1 | 407 |
| F4HQD4 | HSP70-15 | 3702.AT1G79920.1 | Q8RX88 | FACE1 | 3702.AT4G01320.1 | 562 |
| P57106 | MDH2 | 3702.AT5G43330.1 | Q9FN30 | At5g53970 | 3702.AT5G53970.1 | 750 |
| P57106 | MDH2 | 3702.AT5G43330.1 | F4I7I0 | ALAAT1 | 3702.AT1G17290.1 | 778 |
| Q9S9N1 | HSP70-5 | 3702.AT1G16030.1 | Q8RX88 | FACE1 | 3702.AT4G01320.1 | 562 |
| Q9S9N1 | HSP70-5 | 3702.AT1G16030.1 | Q38854 | DXS | 3702.AT4G15560.1 | 824 |
| P50318 | At1g56190 | 3702.AT1G56190.1 | Q94B35 | ISPH | 3702.AT4G34350.1 | 429 |
| Q9SRT9 | RGP1 | 3702.AT3G02230.1 | Q9LY74 | VTE3 | 3702.AT3G63410.1 | 464 |
| Q9SZ30 | HISN4 | 3702.AT4G26900.1 | Q94FY7 | VTE1 | 3702.AT4G32770.1 | 477 |
| P46248 | ASP5 | 3702.AT4G31990.3 | F4I7I0 | ALAAT1 | 3702.AT1G17290.1 | 702 |
| P46248 | ASP5 | 3702.AT4G31990.3 | Q9FN30 | At5g53970 | 3702.AT5G53970.1 | 853 |
| P46248 | ASP5 | 3702.AT4G31990.3 | Q15KI9 | PHYLLO | 3702.AT1G68890.1 | 800 |
| O65282 | CPN20 | 3702.AT5G20720.1 | Q8RX88 | FACE1 | 3702.AT4G01320.1 | 495 |
| F4J2T9 | At3g22980 | 3702.AT3G22980.1 | Q56Y11 | At5g58770 | 3702.AT5G58770.1 | 565 |
| Q8GVE8 | PPC4 | 3702.AT1G68750.1 | F4I7I0 | ALAAT1 | 3702.AT1G17290.1 | 523 |
| Q8GVE8 | PPC4 | 3702.AT1G68750.1 | Q9FN30 | At5g53970 | 3702.AT5G53970.1 | 523 |
| Q9SJD4 | LACS8 | 3702.AT2G04350.1 | Q9M0X9 | 4CLL7 | 3702.AT4G05160.1 | 481 |
| F4JLM5 | At4g10320 | 3702.AT4G10320.1 | P54873 | HMGS | 3702.AT4G11820.2 | 755 |
| F4JLM5 | At4g10320 | 3702.AT4G10320.1 | Q94B35 | ISPH | 3702.AT4G34350.1 | 697 |
| C0Z361 | CPN60B3 | 3702.AT5G56500.1 | Q9XFS9 | DXR | 3702.AT5G62790.2 | 544 |
| C0Z361 | CPN60B3 | 3702.AT5G56500.1 | O81014 | ISPE | 3702.AT2G26930.1 | 554 |
| C0Z361 | CPN60B3 | 3702.AT5G56500.1 | Q9LY74 | MENB | 3702.AT1G60550.1 | 438 |
| C0Z361 | CPN60B3 | 3702.AT5G56500.1 | Q9LY74 | VTE3 | 3702.AT3G63410.1 | 499 |
| Q42601 | CARB | 3702.AT1G29900.1 | P54873 | HMGS | 3702.AT4G11820.2 | 492 |
| Q42601 | CARB | 3702.AT1G29900.1 | Q9FIK7 | At5g47720 | 3702.AT5G47720.2 | 928 |
| Q42601 | CARB | 3702.AT1G29900.1 | Q38854 | DXS | 3702.AT4G15560.1 | 431 |

**Table S3 Expression pattern of prenyltransferase and proteins associated with terpenoid synthesis in *R. glutinosa***

| Uniprot ID | Unigene ID | Protein Name | Fold-Change | | |
| --- | --- | --- | --- | --- | --- |
|  |  |  | I/E | M/E | M/I |
| Q38929 | Unigene9255_RNA | IPP1 | 0.912 | 0.9638 | 1.0471 |
| Q09152 | Unigene5386_RNA | FPS1 | 1.0471 | 1.0864 | 1.028 |
| Q43315 | Unigene75780_RNA | FPS2 | 1.4588 | 1.1169 | 0.7586 |
| Q39108 | Unigene4691_RNA | GGR | 0.7047 | 0.2805 | 0.4169 |
| Q9XFS9 | Unigene10215_RNA | DXR | 2.6303 | 0.5346 | 0.2014 |
| Q38854 | Unigene79781_RNA | DXS | 0.9908 | 0.8872 | 0.8872 |
| F4I7I0 | Unigene75343_RNA | AAT1 | 2.2909 | 1.5136 | 0.6252 |
| Q8RX88 | Unigene20797_RNA | FACE1 | 0.3133 | 0.3802 | 1.2942 |
| Q9LX33 | Unigene6482_RNA | FTA | 0.6982 | 1.3062 | 1.8365 |
| Q76FS5 | Unigene81201_RNA | SPS2 | 0.8954 | 0.863 | 0.9638 |
| Q5HZ00 | Unigene12226_RNA | SPS3 | 0.8166 | 0.8318 | 1.0000 |
| F4JCU3 | Unigene21544_RNA | MVD2 | 1.0568 | 0.9638 | 0.9036 |
| P69834 | Unigene11436_RNA | ISPD | 0.955 | 0.9462 | 0.9817 |
| O81014 | Unigene34464_RNA | ISPE | 1.9409 | 1.0965 | 0.5445 |
| Q9CAK8 | Unigene35334_RNA | ISPF | 1.1695 | 0.9376 | 0.7943 |
| F4K0E8 | Unigene80801_RNA | ISPG | 1.7701 | 1.1169 | 0.6138 |
| Q94B35 | Unigene72841_RNA | ISPH | 0.5445 | 0.5105 | 0.9036 |
| P54873 | Unigene18749_RNA | HMGS | 1.0375 | 0.9462 | 0.8954 |
| Q9CA67 | Unigene19023_RNA | CHLP | 11.9124 | 4.3652 | 0.3631 |
| P34802 | Unigene25249_RNA | GGPPS1 | 1.1376 | 0.7447 | 0.6486 |
| P57681 | Unigene39208_RNA | FCLY | 0.6668 | 0.4656 | 0.6918 |
| Q9M2E2 | Unigene1369_RNA | SDR1 | 0.9204 | 0.929 | 1.0000 |
| Q9ZUH5 | Unigene1776_RNA | SDR2b | 1.0375 | 0.8318 | 0.7943 |
| E3VNM4 | Unigene926_RNA | TGA10 | 0.6792 | 1.1803 | 1.7219 |
| Q9LVY2 | Unigene29746_RNA | PEN3 | 1.9588 | 0.8954 | 0.4529 |
| O49354 | Unigene27063_RNA | COQ3 | 0.9462 | 0.912 | 0.955 |
| Q9LVC8 | Unigene25402_RNA | COQ5 | 0.5105 | 1.028 | 2.0324 |
| F4J6I6 | Unigene13148_RNA | COQ6 | 0.9204 | 0.9727 | 1.0375 |
| Q15KI9 | Unigene19768_RNA | PHYLLO | 1.0186 | 1.0965 | 1.0666 |
| Q8GYN9 | Unigene5910_RNA | MENB | 0.8241 | 0.8241 | 0.9908 |
| Q94FY7 | Unigene13814_RNA | VTE1 | 1.0568 | 1.0093 | 0.9462 |
| Q9LY74 | Unigene14006_RNA | VTE3 | 0.929 | 0.5395 | 0.6138 |
| Q9ZSK1 | Unigene88873_RNA | VTE4 | 1.1066 | 0.9727 | 0.863 |
| P92994 | Unigene48742_RNA | CYP73A5 | 0.8954 | 0.8091 | 0.8954 |
| Q1ACB3 | Unigene11094_RNA | HST | 0.6792 | 0.4207 | 0.5495 |
| Q42524 | Unigene20893_RNA | 4CL1 | 0.787 | 0.9036 | 1.1376 |
| Q9M0X9 | Unigene93108_RNA | 4CLL7 | 0.3076 | 1.2589 | 3.9811 |
| Q9LHR4 | Unigene12015_RNA | At3g32040 | 1.0568 | 0.8551 | 0.8017 |
| Q9LJY2 | Unigene55090_RNA | At3g20160 | 1.3062 | 0.912 | 0.6918 |
| Q9LIA0 | Unigene49477_RNA | At3g29430 | 0.8241 | 0.955 | 1.1482 |
| Q6NQE2 | Unigene70300_RNA | At4g27270 | 0.4285 | 0.6252 | 1.4322 |
| O23207 | Unigene62535_RNA | At4g36750 | 1.028 | 1.0471 | 1.0000 |
| P46086 | Unigene9961_RNA | At5g27450 | 0.7311 | 0.9204 | 1.2474 |
| Q9FIK7 | Unigene78284_RNA | At5g47720 | 1.0471 | 1.0375 | 0.9727 |
| Q9FN30 | Unigene78227_RNA | At5g53970 | 1.0471 | 1.1272 | 1.0666 |
| Q56Y11 | Unigene14062_RNA | At5g58770 | 1.0093 | 0.5058 | 0.492 |
| Q38833 | Unigene1711_RNA | CHLG | 0.9638 | 1.4997 | 1.5276 |
| Q8VYB7 | Unigene77883_RNA | RGTA1 | 2.729 | 2.6303 | 0.955 |
| Q84J75 | Unigene10459_RNA | RGTB1 | 0.6194 | 0.8551 | 1.3677 |
| P37271 | Unigene69078_RNA | PSY1 | 0.9817 | 0.7516 | 0.7586 |
| O80601 | Unigene38410_RNA | T27I1.12 | 1.1066 | 1.5136 | 1.3552 |

E, I, M represents respectively elongation stage, expansion stage, maturation stage of *R. glutinosa*. I/E represents the fold-change of protein expression between I stage and E stage, M/E represents the fold-change of protein expression between M stage and E stage, M/I represents the fold-change of protein expression between M stage and I stage. As the fold-change is greater than 1.2 or less than 0.83, the expressed protein is considered to be differential (p < 0.05).

**Table S4 Some h**[**omologous**](javascript:;)**p**[**rotein**](javascript:;)**s on FPP/GPP synthase family of *R. glutinosa***

| Species | Accession Number | Identity (%) | | | | | | | |
| --- | --- | --- | --- | --- | --- | --- | --- | --- | --- |
|  |  | FPPS | FPPS1 | GGPS | GGP3 | GGPS4 | GGPS5 | GPPS | GPPS2 |
| *Arabidopsis thaliana* | AT4G17190.1 | 77.5 |  |  |  |  |  |  |  |
|  | AT5G47770.1 |  | 80.4 |  |  |  |  |  |  |
|  | AT4G36810.1 |  |  | 73.3 |  |  |  |  |  |
|  | AT3G29430.1 |  |  |  | 69.3 |  |  |  |  |
|  | AT3G14550.1 |  |  |  |  | 71.6 |  |  |  |
|  | AT4G38460.1 |  |  |  |  |  | 66.8 |  |  |
|  | AT2G34630.2 |  |  |  |  |  |  | 68.3 |  |
|  | AT1G17050.1 |  |  |  |  |  |  |  | 69.3 |
| *Vitis vinifera* | VIT_19s0015g01010.t01 | 84.2 |  |  |  |  |  |  |  |
|  | VIT_19s0015g01010.t01 |  | 85.9 |  |  |  |  |  |  |
|  | VIT_04s0023g01210.t01 |  |  | 73.1 |  |  |  |  |  |
|  | VIT_03s0038g03050.t01 |  |  |  | 69.9 |  |  |  |  |
|  | VIT_04s0023g01210.t01 |  |  |  |  | 72.6 |  |  |  |
|  | VIT_03s0038g03050.t01 |  |  |  |  |  | 68.7 |  |  |
|  | VIT_15s0024g00850.t01 |  |  |  |  |  |  | 74.3 |  |
|  | VIT_19s0014g00070.t01 |  |  |  |  |  |  |  | 79.0 |
| *Nicotiana sylvestris* | XP_009759138.1 | 82.5 |  |  |  |  |  |  |  |
|  | XP_009764692.1 |  | 83.0 |  |  |  |  |  |  |
|  | XP_009784771.1 |  |  | 69.0 |  |  |  |  |  |
|  | XP_009788757.1 |  |  |  | 80.0 |  |  |  |  |
|  | XP_009784771.1 |  |  |  |  | 68.4 |  |  |  |
|  | XP_009788757.1 |  |  |  |  |  | 74.5 |  |  |
|  | XP_009793550.1 |  |  |  |  |  |  | 78.7 |  |
|  | XP_009797030.1 |  |  |  |  |  |  |  | 76.5 |
| *Nicotiana tomentosiformis* | XP_009594047.1 | 83.0 |  |  |  |  |  |  |  |
|  | XP_009621499.1 |  | 82.7 |  |  |  |  |  |  |
|  | XP_009627578.1 |  |  | 68.6 |  |  |  |  |  |
|  | XP_009589555.1 |  |  |  | 79.2 |  |  |  |  |
|  | XP_009627578.1 |  |  |  |  | 68.1 |  |  |  |
|  | XP_009589555.1 |  |  |  |  |  | 74.2 |  |  |
|  | XP_009600020.1 |  |  |  |  |  |  | 78.7 |  |
|  | XP_009606871.1 |  |  |  |  |  |  |  | 76.3 |
| *Solanum lycopersicum* | Solyc12g015860.1.1 | 77.5 |  |  |  |  |  |  |  |
|  | Solyc12g015860.1.1 |  | 79.8 |  |  |  |  |  |  |
|  | Solyc02g085700.1.1 |  |  | 69. |  |  |  |  |  |
|  | Solyc09g008920.2.1 |  |  |  | 77.3 |  |  |  |  |
|  | Solyc02g085700.1.1 |  |  |  |  | 68.5 |  |  |  |
|  | Solyc09g008920.2.1 |  |  |  |  |  | 73.8 |  |  |
|  | Solyc08g023470.2.1 |  |  |  |  |  |  | 79.2 |  |
|  | Solyc07g061990.2.1 |  |  |  |  |  |  |  | 73.9 |
| *Cucumis sativus* | XP_004169698.1 | 80.1 |  |  |  |  |  |  |  |
|  | XP_004169698.1 |  | 84.2 |  |  |  |  |  |  |
|  | XP_004164466.1 |  |  | 73.6 |  |  |  |  |  |
|  | XP_004163415.1 |  |  |  | 74.2 |  |  |  |  |
|  | XP_004164466.1 |  |  |  |  | 73.6 |  |  |  |
|  | XP_004163415.1 |  |  |  |  |  | 74.0 |  |  |
|  | XP_004158644.1 |  |  |  |  |  |  | 70.4 |  |
|  | XP_004156502.1 |  |  |  |  |  |  |  | 73.6 |
| *Beta vulgaris* | XP_010675977.1 | 78.4 |  |  |  |  |  |  |  |
|  | XP_010675977.1 |  | 85.0 |  |  |  |  |  |  |
|  | XP_010680905.1 |  |  | 75.9 |  |  |  |  |  |
|  | XP_010690298.1 |  |  |  | 72.1 |  |  |  |  |
|  | XP_010680905.1 |  |  |  |  | 75.9 |  |  |  |
|  | XP_010690298.1 |  |  |  |  |  | 70.8 |  |  |
|  | XP_010670594.1 |  |  |  |  |  |  | 71.7 |  |
|  | XP_010684494.1 |  |  |  |  |  |  |  | 73.6 |
| *Erythranthe guttata* | Migut.H01742.1.p | 90.9 |  |  |  |  |  |  |  |
|  | Migut.H01742.1.p |  | 80.8 |  |  |  |  |  |  |
|  | Migut.H00196.1.p |  |  | 69.1 |  |  |  |  |  |
|  | Migut.N01338.1.p |  |  |  | 87.8 |  |  |  |  |
|  | Migut.H00196.1.p |  |  |  |  | 69.1 |  |  |  |
|  | Migut.N01338.1.p |  |  |  |  |  | 79.9 |  |  |
|  | Migut.G00061.1.p |  |  |  |  |  |  | 88.2 |  |
|  | Migut.H02035.1.p |  |  |  |  |  |  |  | 86.9 |

The h[omologs](javascript:;) for FPPS, FPPS1, GGPS, GGP3, GGPS4, GGPS5, GPPS, SPS2 of *R. glutinosa* were searched from 76 species of plants, and were all obtained from 39 species of plants. The h[omologs](javascript:;) in the above 8 species had high similarity with FPP/GPP synthase family of *R. glutinosa.*


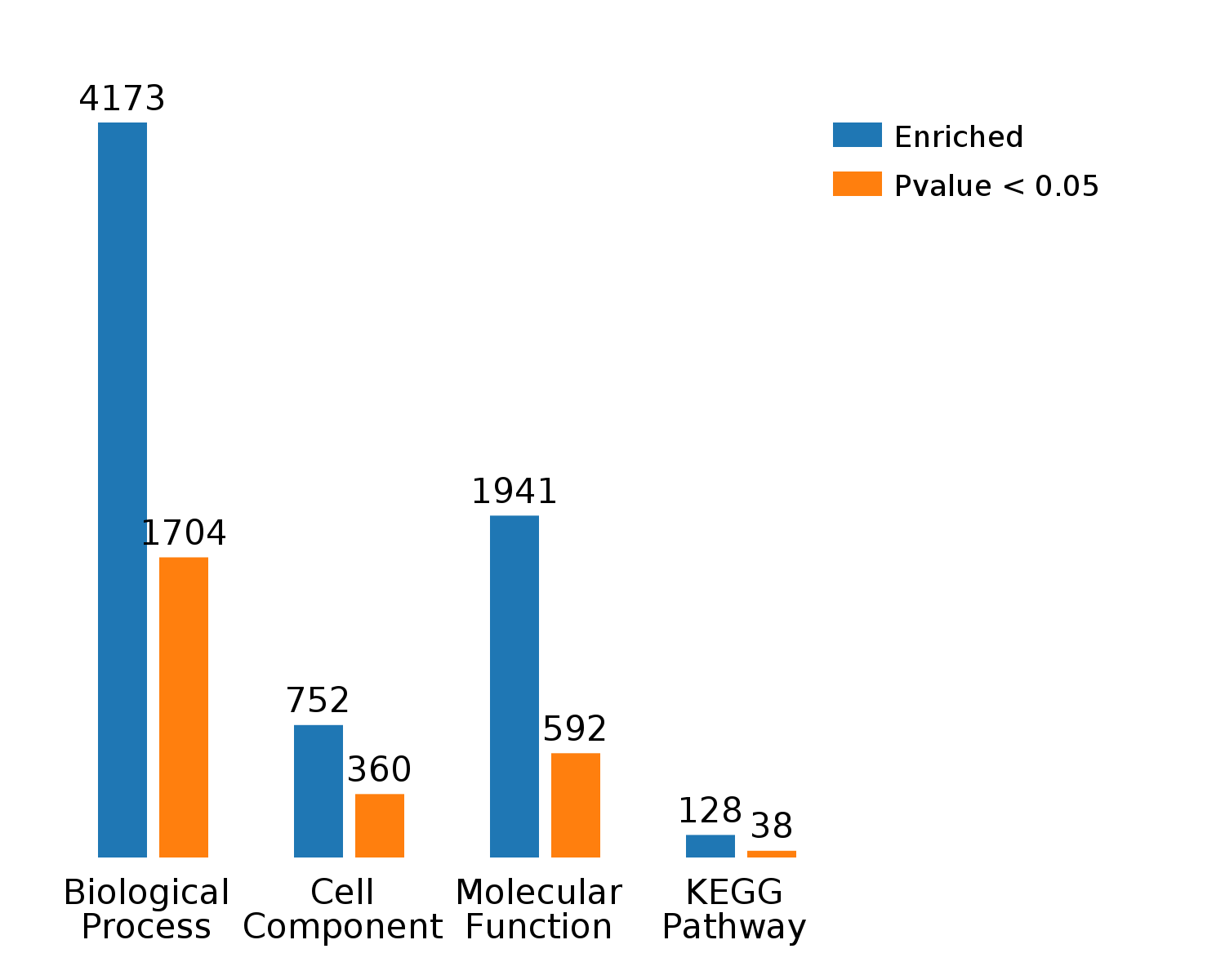


**Fig. S1 Results of bioinformatics function analysis of proteins in *R. glutinosa***

(a)
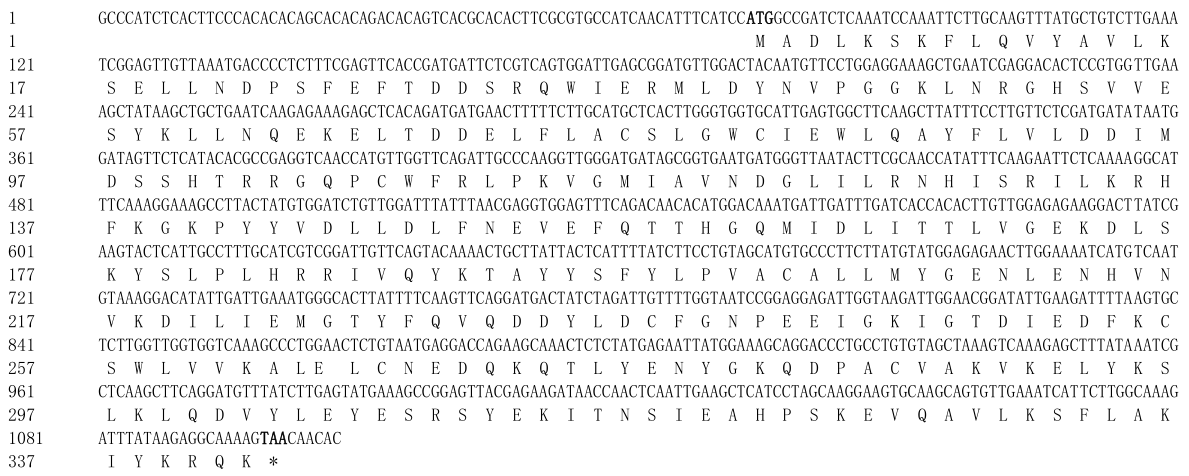


(b)
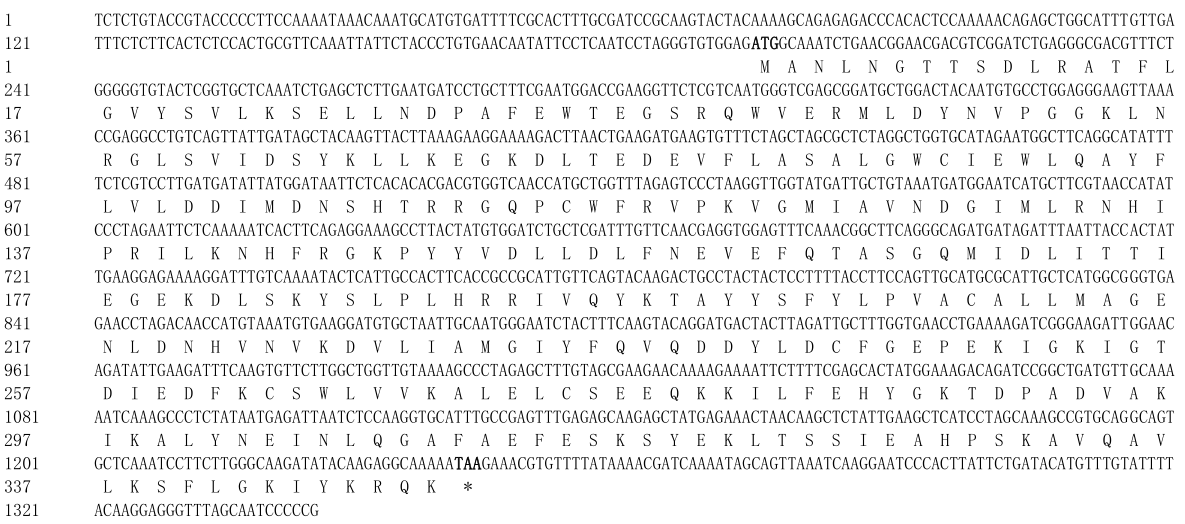


(c)
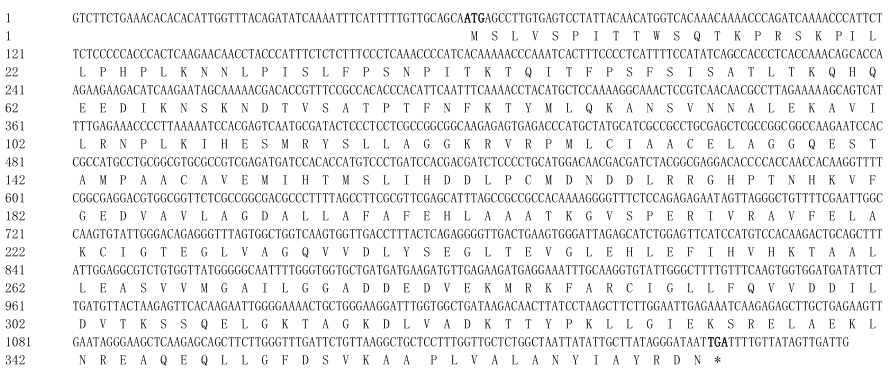


(d)
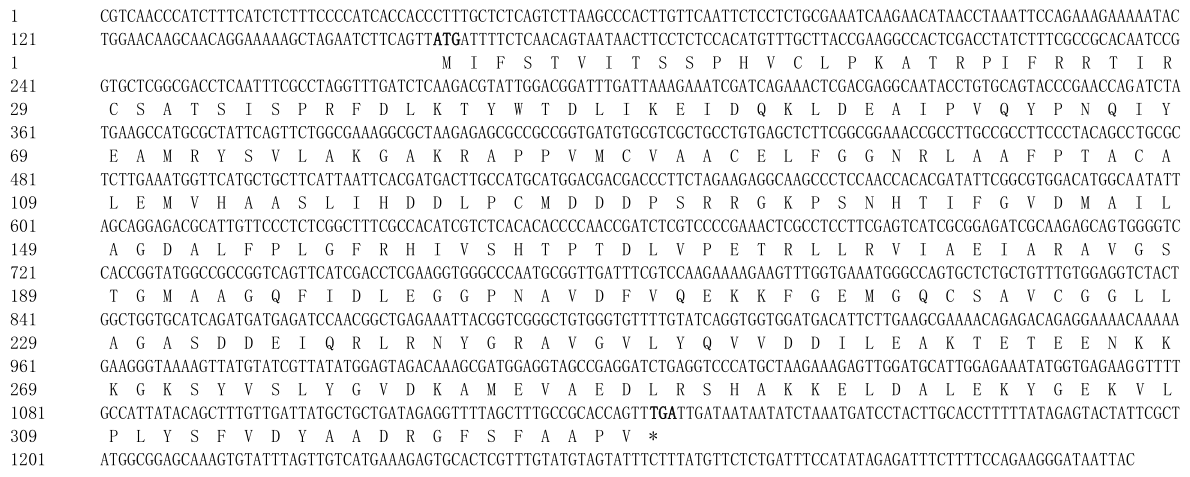


(e)
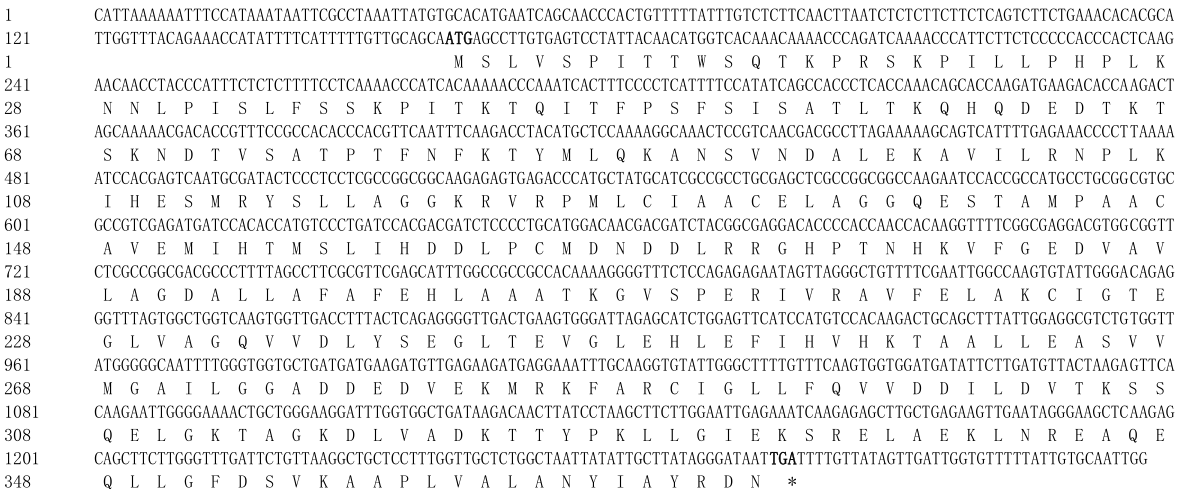


(f)
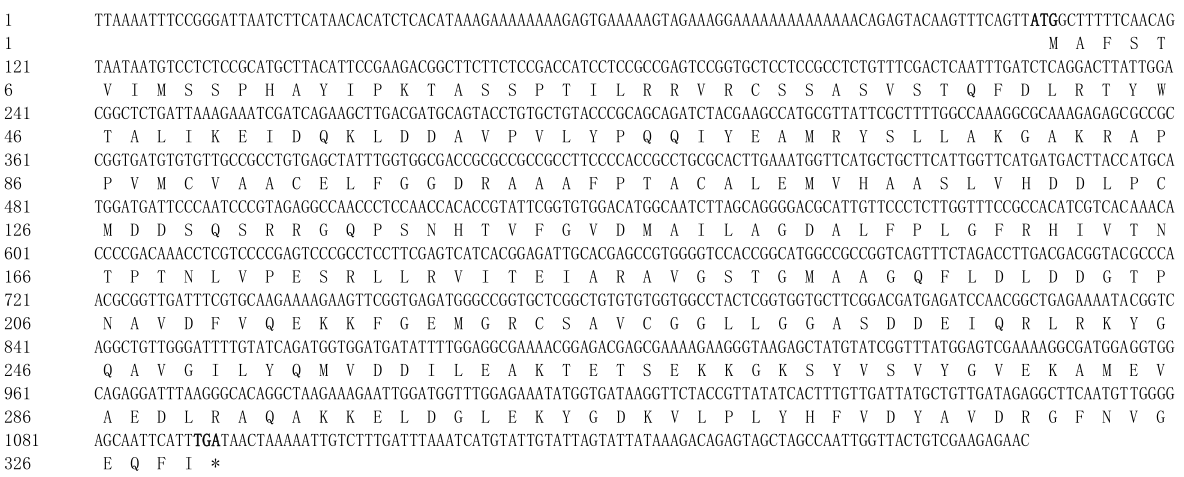


(g)
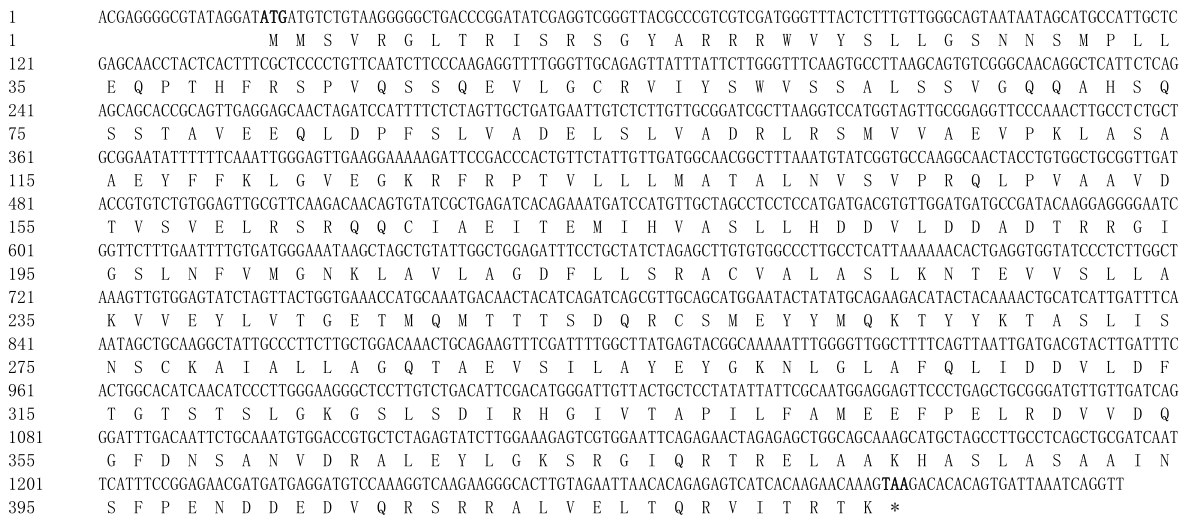


(h)
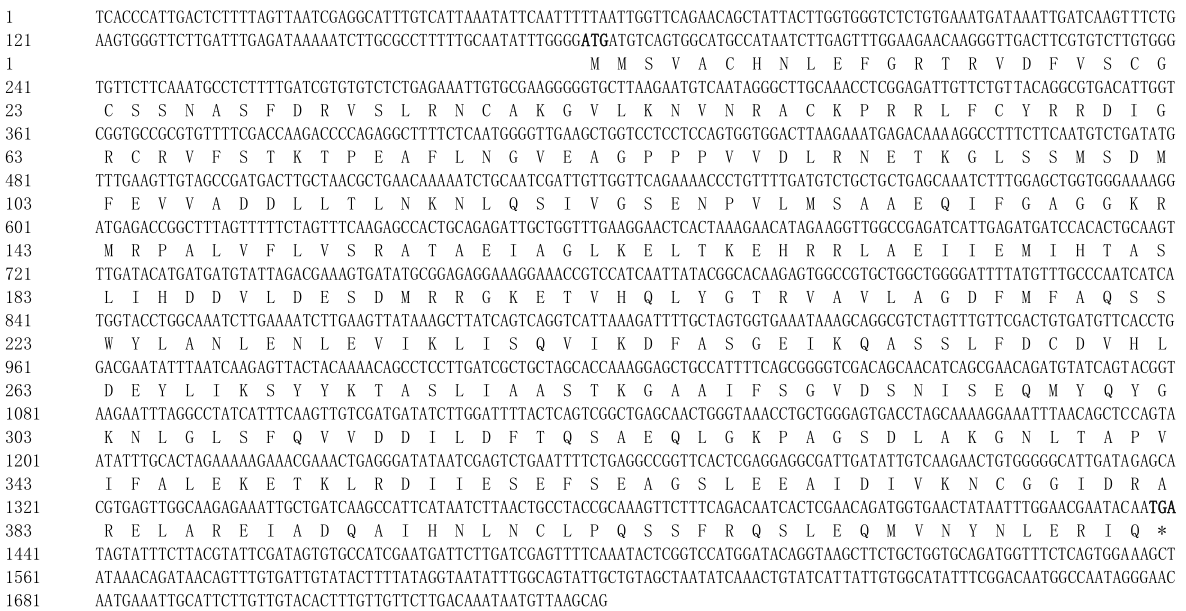


**Fig. S2 The cDNA and amino acid sequences of FPP/GGPP synthase in *R.glutinosa***

(a), (b), (c), (d), (e), (f), (g) and (h) represents the CDS and amino acid sequences of *FPPS* (MG770217), *FPPS1* (MT680921), *GGPS* (MG770218), *GGPS3* (MT680922), *GGPS4* (MT680923), *GGPS5* (MW298275), *GPPS* (MG770219) and *GPPS2* (MW656184), respectively.

(a)
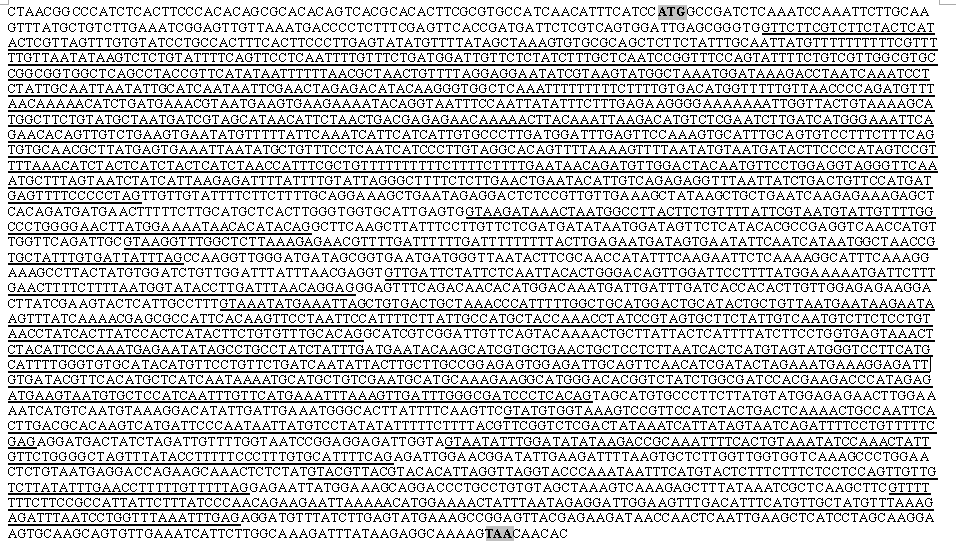


(b)
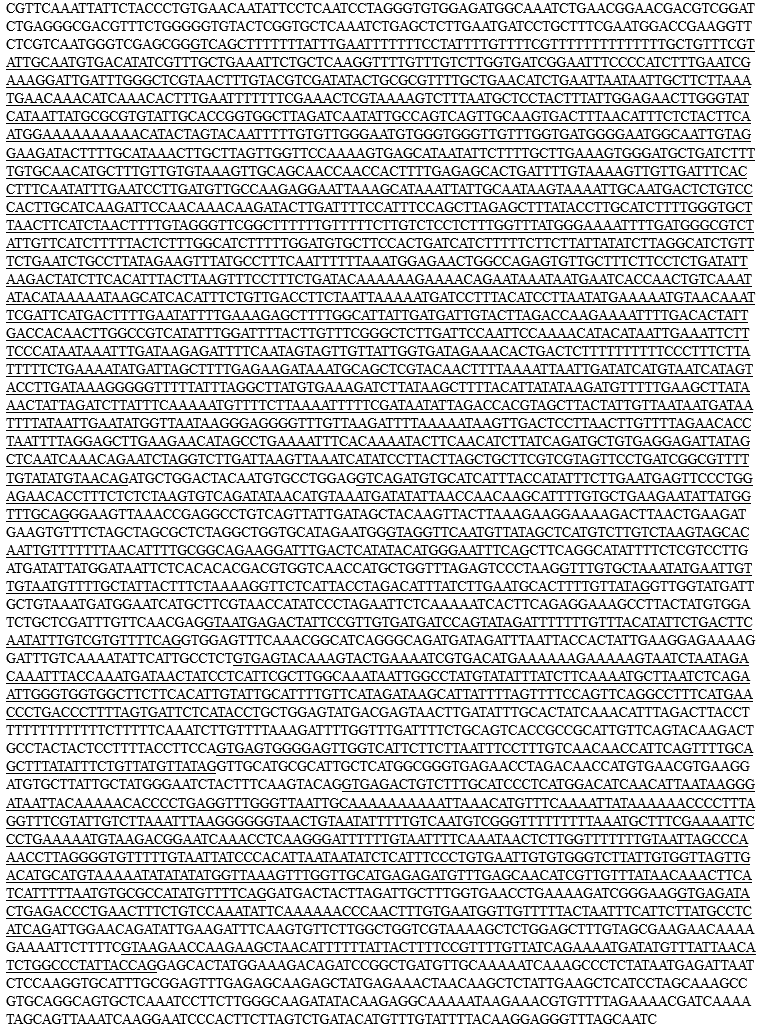


(c)
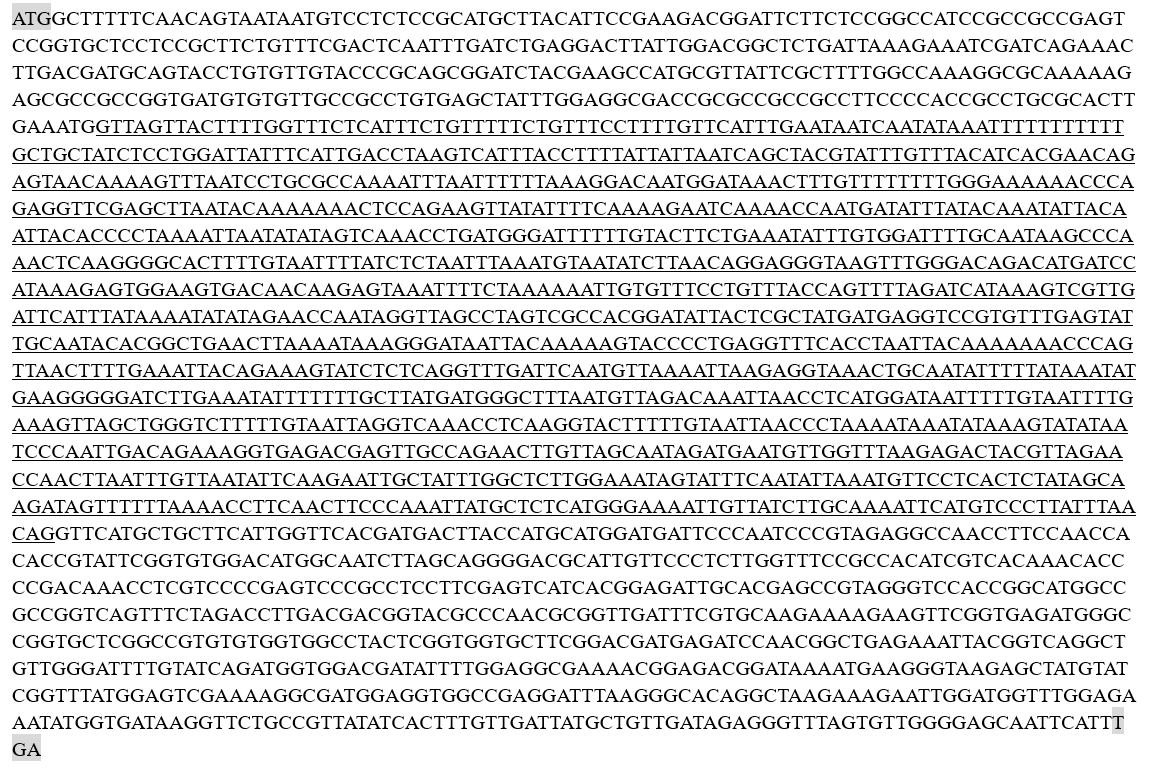


(d)
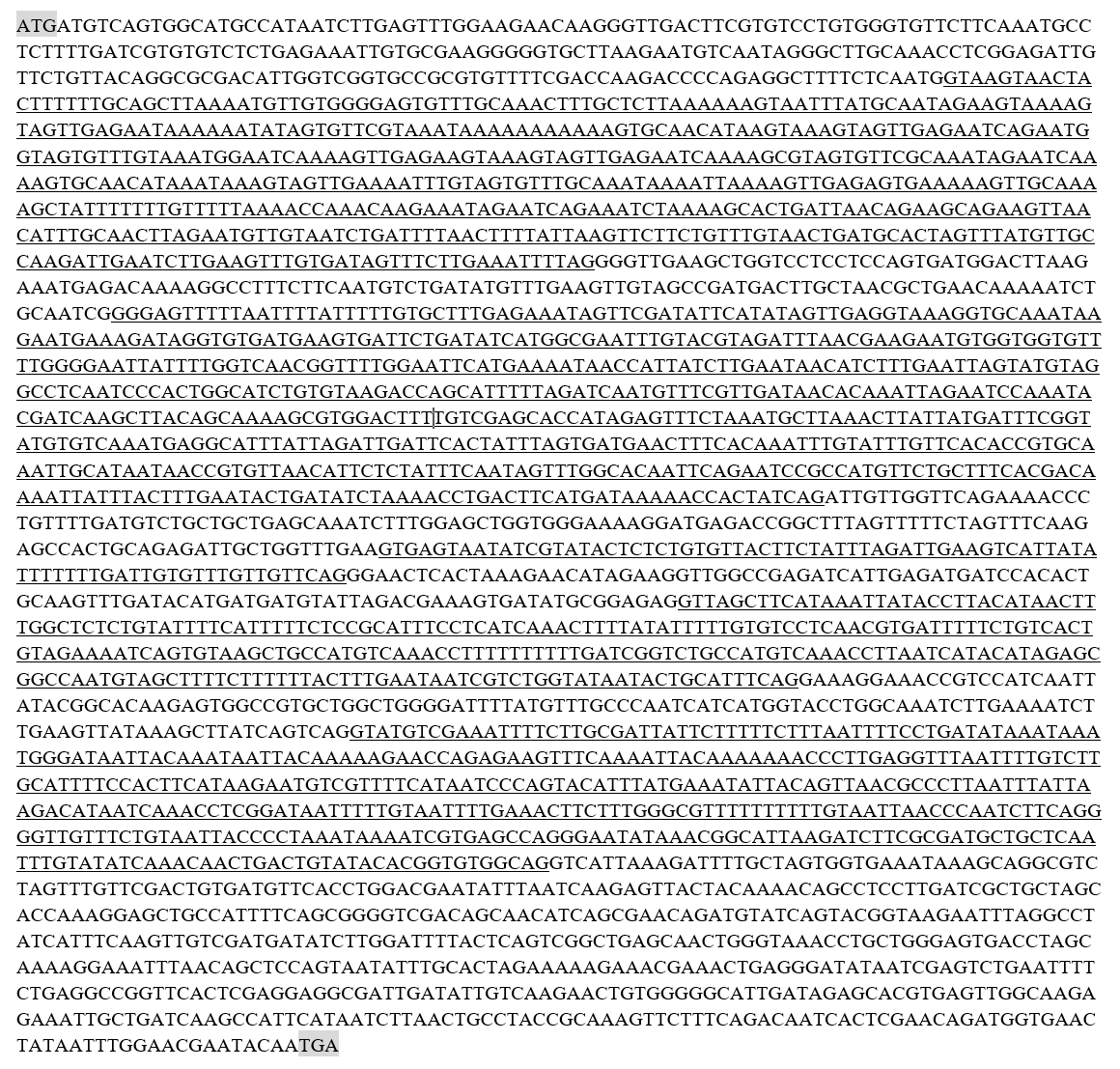


**Fig. S3 The DNA sequences of some FPP/GGPP synthase in *R. glutinosa***

(a), (b), (c) and (d) represents DNA sequences of *FPPS, FPPS1, GGPS5, GPPS2*, respectively, and the intron sequences are shown with the underlined regions.
